# Privileged fragment-based design, synthesis and in vitro antitumor activity of imatinib analogues

**DOI:** 10.55730/1300-0527.3549

**Published:** 2023-02-14

**Authors:** Hongyu JIANG, Yuankun WANG, Maokai JIANG, Lei YAO

**Affiliations:** School of Pharmacy, Collaborative Innovation Center of Advanced Drug Delivery System and Biotech Drugs in Universities of Shan-dong, Key Laboratory of Molecular Pharmacology and Drug Evaluation (Yantai University), Ministry of Education, Yantai University, Yantai, China

**Keywords:** Privileged fragment-based drug design, imatinib, antitumor, synthesis

## Abstract

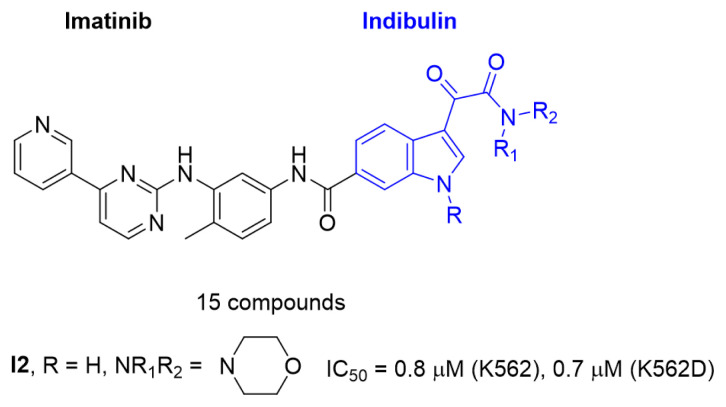

Based on the privileged fragment-based drug design strategy, a series of imatinib analogues bearing the moiety of 3-(2-amino-2-oxoacetyl)-1H-indole were designed and synthesized, and the in vitro antitumor activity of these compounds was detected by MTT method using K562 (human myeloid leukemia) and K562R (imatinib-resistant chronic myeloid leukemia) cell lines. Molecular docking was used to preliminarily explain the possible binding modes. The most potent compound **I2** exhibited better antitumor activity than those of imatinib against K562 and K562R cancer cells with IC_50_ values of 0.8 μM and 0.7 μM.

## 1. Introduction

With the development of molecular biochemistry and related subjects, the possible mechanism of oncogenesis and development is gradually revealed. Due to the important roles protein tyrosine kinases (PTKs) play in tumorigenesis and progression of some human cancers, medicinal chemists all over the world devoted to discovering selective tyrosine kinases inhibitors (TKIs) [1,2].

Imatinib, a selective Bcr-Abl tyrosine kinase inhibitor developed by Novartis, was approved by FDA in 2001 for the treatment of chronic myelogenous leukemia (CML) [3,4]. It could inhibit the enzyme activity by competitively binding to the ATP-binding site of the kinase, and subsequently preventing phosphorylation and blocking the downstream signal transduction pathways. The success of imatinib was once recognized as the miracle breakthrough in the human anticancer history [5].

**Figure 1:**
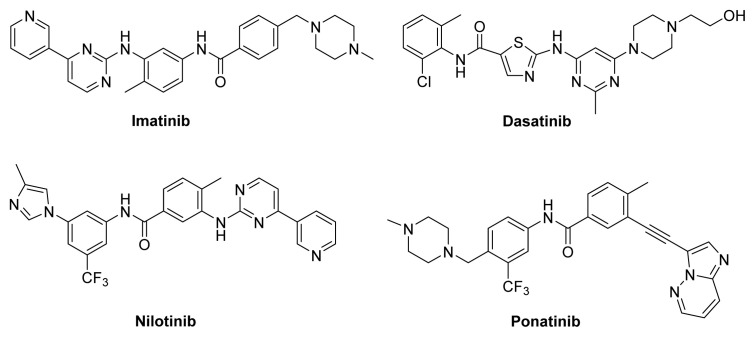
The structures of Bcr-Abl kinase inhibitors.

Due to the reactivation or mutation of Bcr-Abl kinase, drug resistance occurred for certain patients [6]. To overcome the drug resistance, the second generation of Bcr-Abl kinase inhibitors, such as nilotinib and dasatinib (Figure 1), were developed, and they exhibited better inhibitory activity against wild-typed and mutant Bcr-Abl kinases than imatinib [7]. However, they were still not effective to T315I mutation [8,9]. Ponatinib, the third generation Bcr-Abl kinase inhibitor, is highly effective to T315I mutation. The severe side-effects including cardiovascular thrombotic events due to the off-target effect is still a critical issue [10,11]. Therefore, the searching for more potent and less toxic anticancer PTKs is always attractive to medicinal chemists.

**Figure 2:**
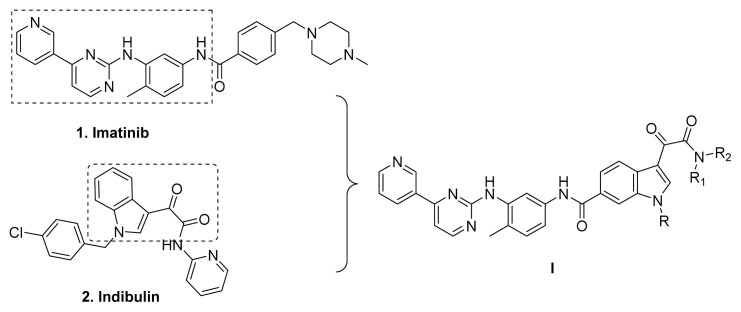
The design of target compound I.

Indibulin (Figure 2), a microtubule inhibitor, exhibited potent activity towards a wide variety of cancer cell lines including multidrug-resistant cells lines. It showed no apparent toxicity at curative doses and was well tolerated in human patients [12]. Based on the privileged fragment-based drug design strategy a series of compounds with structural formula **I** were designed [13, 14], in which the indolglyoxylamide moiety from indibulin was introduced into the structure of imatinib to replace the hydrophobic piperazine-benzoic acid part [15,16]. Hopefully, the indolglyoxylamide moiety would provide not only hydrophobic properties, but also certain antitumor activity.

## 2. Results and discussion

### 2.1. Chemistry

**Scheme 1:**
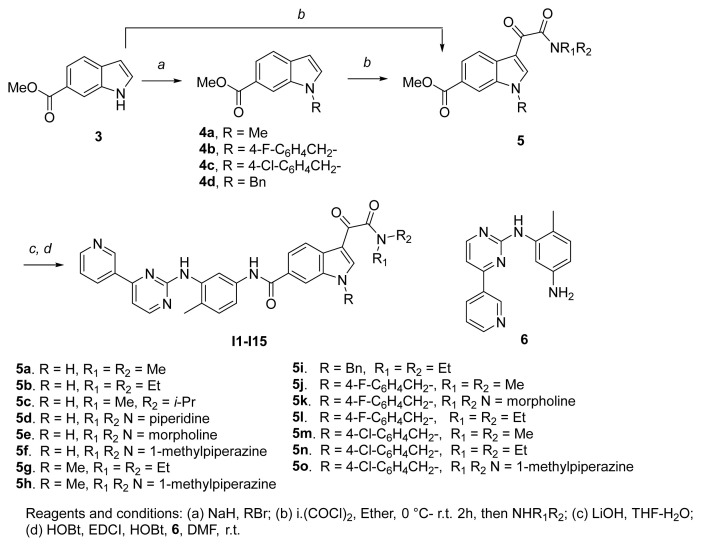
Synthetic route of target compounds **I1**–**I15**.

The synthesis of the compounds **I1**–**I15** was accomplished by a four-step transformation from commercially available materials (Scheme 1). Firstly, methyl indole-6-carboxylate (**3**) was alkylated to generated intermediate **4**, which was then treated with oxalyl chloride and subsequently with appropriate amines to form amide compounds **5g–5o**. For intermediates **5a–5f**, they were obtained directly from compound **3** by the above same procedure. The ester group in **5** was hydrolyzed to the corresponding carboxylic acid, and then an amide formation reaction with amine compound **6** under classical amide condensation condition afforded the target compound **I1**–**I15** in moderate yields. Compound **6** was prepared according to the reported methods [17, 18].

### 2.2. In vitro antitumor activity assay

The antitumor activity of all synthesized compounds (**I1–I15**) was tested on the K562 (chronic myelogenous leukemia) and K562R (imatinib-resistant) cell lines, using the 3-(4,5-dimethylthiazolyl-2)-2,5-diphenyltetrazolium bromide (MTT) assay. The results were expressed as IC_50_ values and summarized in Table.

All the synthesized compounds showed certain antitumor activity with the IC_50_ values ranging from 0.7 μM to 15 μM against both K562 and K562R cell lines. The most potent compound **I2** showed better antitumor activity than that of imatinib with the IC_50_ values of 0.8 μM and 0.7μM, respectively. A preliminary study of the structure-activity relationship of these derivatives was summarized as follows: 1. Compounds with substitution (**I7**–**I15**) on the indole-nitrogen generally possessed less antitumoral activity. For example, compound **I1** (IC_50_ = 4.1 and 7.6 μM) was more potent than compound **I10** (IC_50_ = 9.7 and 14.7μM) and compound **I13** (IC_50_ =17.8 and 15.9 μM); compound **I6** (IC_50_ = 5.2 and 4.0 μM) was more potent than compound **I8** (IC_50_ = 16.1 and 12.4 μM) and compound **I15** (IC_50_ = 22.5 and 22.8 μM). The possible reason was that the bulky group prevented the molecule from entering into the binding pocket of kinase. 2. The side hydrophobic chain (NR_1_R_2_) had no apparent effect on the antitumor activity. For example, the antitumor activity of compounds **I1–I6** was at the same single digital level. These results suggested that this part was lingering out the binding domain, and it could be tolerant of much modification to obtain drug candidates with appropriate pharmacodynamic/pharmacokinetic (PD/PK) properties. Compound **I2** was also tested on the WSS-1 cells to check the selectivity. It showed less toxicity to WSS-1 cell line with the IC_50_ value of 78.6 ± 17.2 μM. Compound **I2** may represent a promising lead for the further development of novel antitumor drugs.

### 2.3. Molecular docking study

**Figure 3:**
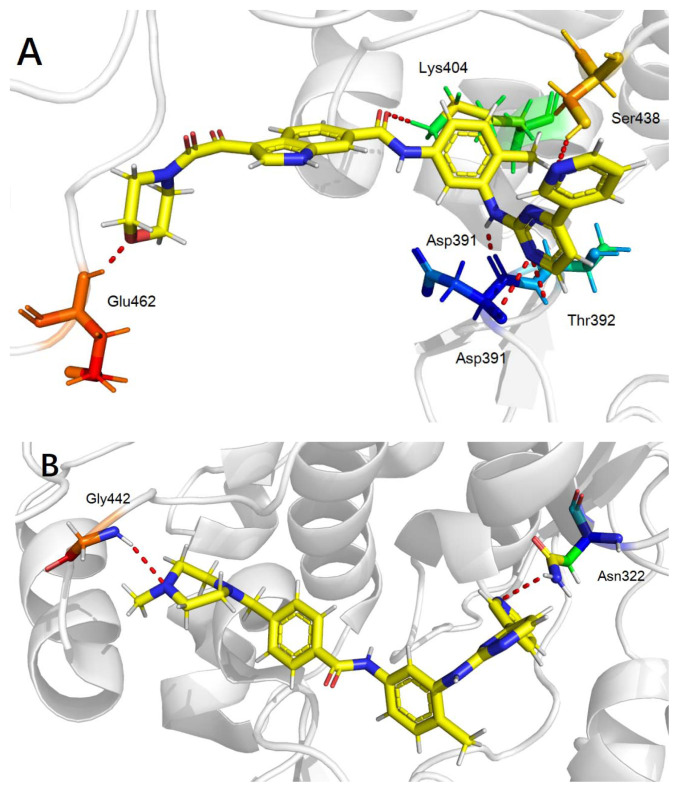
The molecular docking results. A is predicted binding model of compound **I2** with 1IEP, and B is predicted binding model of imatinib with 1IEP. H-bonds are shown by dashed lines.

To gain an understanding of how these compounds interact with the target proteins, and more importantly, to direct our optimization strategy, a molecular docking study was performed using the cocrystal structure of imatinib and Bcr-Abl kinase (PDB code: 1IEP) by Sybyl 2.1.1 software [19]. Imatinib and the most potent compound **I2** were selected for the molecular docking study, according to the general protocol. The predicted binding mode was shown in Figure 3, which could partially explain the reason for the high potency of compound **I2**. The phenylpyrimidinamine moiety of both imatinib and compound **I2** had H-bond interaction with the kinase, which acted as an anchor force to set the molecular configuration. There were five H-bond interactions for compound **I2** within the kinase ATP-binding domain with residue Asp391, Thr392, Lys404 and Ser438, and one H-bond interaction with Glu462 residue at the hydrophobic loop. The hydrogen bond distances were 2.1 Å, 2.8 Å, 2.4 Å, 1.8 Å, and 2.1 Å, respectively. However, only two H-bond interactions formed between imatinib and the kinase. The hydrogen bond distances were 2.1 Å and 2.3 Å. Moreover, the interaction of the morpholine group of the compound **I2** gave us a direction for further optimization.

### 2.4. Conclusion

The privileged fragment hybridization strategy is a classic drug design method. It has advantages including improving the biological activity and pharmacokinetic properties of the compound. Based on this strategy, a series of imatinib analogues bearing the moiety of 3-(2-amino-2-oxoacetyl)-1H-indole were designed and synthesized, and the in vitro antitumor activity of these compounds was detected by MTT method. Molecular docking was used to preliminarily explain the possible binding modes. The most potent compound **I2** exhibited better antitumor activity than those of imatinib against K562 and K562R cancer cells with the IC_50_ value of 0.8 μM and 0.7 μM. The preliminary structure-activity relationship established in the present study has laid the foundation for the subsequent structural optimizations, for example the synthesis and activity investigation of I2 analogues with benzyl moieties as the R group. The results for such studies will be reported in due course.

## 3. Experiments section

### 3.1. General methods

All high-quality reagents were commercially available and used without further purification unless otherwise noted. The NMR spectra of the intermediates and final products in a deuterated solvent were detected on a Bruker 400MHz spectrometer. Melting points (MP) were recorded on the WRR melting point apparatus and are uncorrected.

### 3.2. General procedure for the preparation of compound I1-I15

#### 3-(2-(Dimethylamino)-2-oxoacetyl)-*N*-(4-methyl-3-((4-(pyridin-3-yl)pyrimidin-2-yl)amino)phenyl)-1*H*-indole-6-carboxamide (I1)

To a cold solution of compound **5a** (supporting information) (0.10 g, 0.38 mmol) in DMF (3.0 mL), was added EDCI (0.11 g, 0.58 mmol) and HOBt (0.052 g, 0.384 mmol) subsequently. The reaction mixture was allowed to stir at room temperature for 1 h, then a solution of compound **6** (0.11 g, 0.38 mmol) in DMF (7.0 mL) was added. The resulting reaction mixture was stirred at room temperature for 12 h till TLC showed that all starting material consumed. The reaction mixture was diluted with water, extracted with ethyl acetate twice. The combined organic layers was washed with brine, died, filtered, and concentrated. The residue was purified by a flash chromatography (MeOH:DCM, 1:50 to 1:20, V/V) to afford the title compound as white solid, yield: 42%, Mp: 232.1–234.0 °C. ^1^H NMR (400 MHz, DMSO-*d*_6_) *δ* 12.60 (d, *J* = 3.0 Hz, 1H), 10.25 (s, 1H), 9.26 (d, *J* = 1.7 Hz, 1H), 8.95 (s, 1H), 8.66 (dd, *J* = 4.8, 1.6 Hz, 1H), 8.49 (d, *J* = 5.1 Hz, 1H), 8.47 (dt, *J* = 8.0, 1.8 Hz, 1H), 8.26 (d, *J* = 3.3 Hz, 1H), 8.16 (d, *J* = 8.4 Hz, 1H), 8.14–8.13 (m, 1H), 8.10 (d, *J* = 2.1 Hz, 1H), 7.88 (dd, *J* = 8.4, 1.6 Hz, 1H), 7.52–7.47 (m, 2H), 7.41 (d, *J* = 5.2 Hz, 1H), 7.20 (d, *J* = 8.6 Hz, 1H), 2.98 (s, 3H), 2.91 (s, 3H), 2.21 (s, 3H); ^13^C NMR (100 MHz, DMSO-*d*_6_) *δ* 186.6, 167.1, 165.6, 161.6, 161.2, 159.5, 151.4, 148.2, 139.1, 137.8, 137.4, 136.4, 134.5, 132.3, 130.4, 130.0, 127.6, 127.3, 123.8, 122.0, 120.4, 117.3, 116.9, 113.0, 112.7, 107.5, 36.8, 33.5, 17.7; HRMS (*m/z*): [M+H]^+^ calcd for C_29_H_25_N_7_O_3_: 520.2092, found: 520.2095.

#### *N*-(4-Methyl-3-((4-(pyridin-3-yl)pyrimidin-2-yl)amino)phenyl)-3-(2-morpholino-2-oxoacetyl)-1*H*-indole-6-carboxamide (I2)

White solid, 65.3 mg, yield: 35.2%, Mp: 228.7–230.6 °C. ^1^H NMR (400 MHz, DMSO-*d*_6_) *δ* 12.66 (s, 1H), 10.28 (s, 1H), 9.28 (s, 1H), 8.98 (s, 1H), 8.68 (dd, *J* = 4.7, 1.5 Hz, 1H), 8.52 (d, *J* = 5.1 Hz, 1H), 8.49 (dt, *J* = 8.0, 1.9 Hz, 1H), 8.38 (d, *J* = 2.5 Hz, 1H), 8.20 (d, *J* = 8.4 Hz, 1H), 8.17 (s, 1H), 8.12 (d, *J* = 2.0 Hz, 1H), 7.91 (dd, *J* = 8.4, 1.5 Hz, 1H), 7.54–7.49 (m, 2H), 7.43 (d, *J* = 5.2 Hz, 1H), 7.22 (d, *J* = 8.5 Hz, 1H), 3.74–3.69 (m, 2H), 3.67–3.61 (m, 2H), 3.56–3.52 (m, 2H), 3.39–3.36 (m, 2H), 2.23 (s, 3H); ^13^C NMR (100 MHz, DMSO-*d*_6_) *δ* 186.0, 165.7, 165.5, 161.6, 161.2, 159.5, 151.4, 148.2, 137.8, 137.3, 136.5, 134.4, 132.2, 130.4, 130.0, 128.7, 127.6, 127.3, 123.8, 122.0, 120.4, 117.3, 116.8, 113.2, 112.8, 107.5, 66.2, 46.0, 17.7; HRMS (*m/z*): [M+H]^+^ calcd for C_31_H_27_N_7_O_4_: 562.2197, found 562.2195.

#### *N*-(4-Methyl-3-((4-(pyridin-3-yl)pyrimidin-2-yl)amino)phenyl)-3-(2-oxo-2-(piperidin-1-yl)acetyl)-1*H*-indole-6-carboxamide (I3)

White solid, 63.1 mg, yield:33.9%, Mp: 216.1–218.0 °C; ^1^H NMR (400 MHz, DMSO-*d*_6_) *δ* 12.58 (s, 1H), 10.26 (s, 1H), 9.26 (s, 1H), 8.96 (s, 1H), 8.66 (d, *J* = 3.4 Hz, 1H), 8.50 (d, *J* = 5.1 Hz, 1H), 8.47 (dt, *J* = 8.0, 1.9 Hz, 1H), 8.29 (d, *J* = 2.6 Hz, 1H), 8.19–8.13 (m, 2H), 8.10 (s, 1H), 7.89 (d, *J* = 8.4 Hz, 1H), 7.52–7.48 (m, 2H), 7.41 (d, *J* = 5.2 Hz, 1H), 7.20 (d, *J* = 8.4 Hz, 1H), 3.57 (s, 2H), 3.28 (t, *J* = 5.4 Hz, 2H), 2.22 (s, 3H), 1.60 (s, 4H), 1.42 (s, 2H); ^13^C NMR (100 MHz, DMSO-*d*_6_) *δ* 186.7, 165.6, 165.5, 161.6, 161.2, 159.5, 151.4, 148.2, 138.9, 137.8, 137.3, 136.5, 134.4, 132.2, 130.4, 130.0, 127.6, 127.2, 123.8, 121.9, 120.4, 117.3, 116.8, 113.2, 112.8, 107.5, 46.5, 25.9, 25.1, 23.9, 17.7; HRMS (*m/z*): [M+H]^+^ calcd for C_29_H_25_N_7_O_3_: 560.2405, found:560.2406.

#### 3-(2-(Diethylamino)-2-oxoacetyl)-*N*-(4-methyl-3-((4-(pyridin-3-yl)pyrimidin-2-yl)amino)phenyl)-1*H*-indole-6-carboxamide (I4)

Yellow solid, 80.1 mg, yield: 44.2%, Mp: 227.8–229.7 °C; ^1^H NMR (400 MHz, DMSO-*d*_6_) δ 12.56 (s, 1H), 10.25 (s, 1H), 9.26 (s, 1H), 8.96 (s, 1H), 8.66 (dd, *J* = 4.7, 1.4 Hz, 1H), 8.49 (d, *J* = 5.1 Hz, 1H), 8.46 (dt, *J* = 8.0, 1.7 Hz, 1H), 8.21 (d, *J* = 2.9 Hz, 1H), 8.17–8.13 (m, 2H), 8.10 (d, *J* = 2.1 Hz, 1H), 7.88 (dd, *J* = 8.3, 1.6 Hz, 1H), 7.51–7.47 (m, 2H), 7.40 (d, *J* = 5.2 Hz, 1H), 7.20 (d, *J* = 8.6 Hz, 1H), 3.43 (q, *J* = 7.1 Hz, 2H), 3.24 (q, *J* = 7.0 Hz, 2H), 2.21 (s, 3H), 1.17 (t, *J* = 7.1 Hz, 3H), 1.06 (t, *J* = 7.0 Hz, 3H); ^13^C NMR (100 MHz, DMSO-*d*_6_) *δ* 186.6, 166.9, 165.6, 161.6, 161.2, 159.5, 151.4, 148.2, 138.7, 137.8, 137.4, 136.4, 134.4, 132.2, 130.4, 130.0, 127.6, 127.3, 123.8, 121.9, 120.4, 117.3, 116.9, 113.1, 112.8, 107.5, 41.7, 38.2, 17.7, 14.2, 12.7; HRMS (*m/z*): [M+H]^+^ calcd for C_29_H_25_N_7_O_3_: 548.2405, found:548.2403.

#### 3-(2-(Isopropyl(methyl)amino)-2-oxoacetyl)-*N*-(4-methyl-3-((4-(pyridin-3-yl)pyrimidin-2-yl)amino)phenyl)-1*H-*indole-6-carboxamide (I5)

White solid, 88.7 mg, yield: 46.7%, Mp: 232.6–234.5 °C; ^1^H NMR (400 MHz, DMSO-*d*_6_) *δ* 12.63 (s, 1H), 10.29 (s, 1H), 9.29 (dd, *J* = 2.3, 0.8 Hz, 1H), 9.01 (s, 1H), 8.68 (dd, *J* = 4.8, 1.7 Hz, 1H), 8.52 (d, *J* = 5.1 Hz, 1H), 8.50–8.47 (m, 1H), 8.33–8.23 (m, 1H), 8.19–8.16 (m, 2H), 8.13 (d, *J* = 1.8 Hz, 1H), 7.91 (d, *J* = 8.4 Hz, 1H), 7.54–7.50 (m, 2H), 7.43 (d, *J* = 5.2 Hz, 1H), 7.22 (d, *J* = 8.7 Hz, 1H), 3.88–3.82 (m, 1H), 2.87–2.77 (m, 3H), 2.23 (s, 3H), 1.20–1.12 (m, 6H); ^13^C NMR (100 MHz, DMSO-*d*_6_) *δ* 186.9, 167.0, 166.8, 165.6, 161.6, 161.2, 159.5, 151.4, 148.2, 137.8, 137.4, 136.5, 134.5, 132.3, 130.4, 130.1, 127.6, 127.2, 123.8, 121.9, 120.4, 117.3, 116.8, 113.2, 112.8, 107.5, 55.0, 24.7, 19.8, 18.8, 17.7; HRMS (*m/z*): [M+H]^+^ calcd for C_29_H_25_N_7_O_3_: 548.2405, found: 548.2410.

#### *N*-(4-Methyl-3-((4-(pyridin-3-yl)pyrimidin-2-yl)amino)phenyl)-3-(2-(4-methylpiperazin-1-yl)-2-oxoacetyl)-1*H-*indole-6-carboxamide (I6)

Yellow solid, 77.6 mg, yield: 42.6%, Mp: 234.5–237.4 °C; ^1^H NMR (400 MHz, DMSO-*d*_6_) *δ* 12.70 (s, 1H), 10.29 (s, 1H), 9.28 (d, *J* = 1.7 Hz, 1H), 8.98 (s, 1H), 8.68 (dd, *J* = 4.8, 1.6 Hz, 1H), 8.51 (d, *J* = 5.1 Hz, 1H), 8.50–8.46 (m, 1H), 8.32 (s, 1H), 8.21–8.17 (m, 2H), 8.13 (d, *J* = 2.0 Hz, 1H), 7.92 (dd, *J* = 8.4, 1.6 Hz, 1H), 7.54–7.50 (m, 2H), 7.42 (d, *J* = 5.2 Hz, 1H), 7.22 (d, *J* = 8.6 Hz, 1H), 3.64 (t, *J* = 4.8 Hz, 2H), 3.35 (t, *J* = 5.0 Hz, 2H), 2.44 (t, *J* = 4.9 Hz, 2H), 2.29 (t, *J* = 5.0 Hz, 2H), 2.22 (d, *J* = 8.8 Hz, 6H); ^13^C NMR (100 MHz, DMSO-*d*_6_) *δ* 186.2, 165.6, 161.6, 161.2, 159.5, 151.4, 148.2, 137.8, 137.4, 136.5, 134.5, 132.3, 130.4, 130.1, 127.6, 127.3, 123.8, 122.0, 120.4, 118.8, 117.3, 116.9, 113.2, 112.8, 110.1, 107.5, 62.8, 56.1, 54.5, 53.9, 45.5, 17.7; HRMS (*m/z*): [M+H]^+^ calcd for C_29_H_25_N_7_O_3_: 575.2514, found: 575.2510.

#### 3-(2-(Diethylamino)-2-oxoacetyl)-1-methyl-*N*-(4-methyl-3-((4-(pyridin-3-yl)pyrimidin-2-yl)amino)phenyl)-1*H-*indole-6-carboxamide (I7)

White solid, 114.2 mg, yield: 61.5%, Mp: 230.3–32.1°C; ^1^H NMR (400 MHz, DMSO-*d*_6_) *δ* 10.25 (s, 1H), 9.29 (s, 1H), 8.99 (s, 1H), 8.68 (d, *J* = 4.0 Hz, 1H), 8.52 (d, *J* = 5.1 Hz, 1H), 8.48 (d, *J* = 7.8 Hz, 1H), 8.29 (d, *J* = 10.0 Hz, 2H), 8.20 (d, *J* = 8.2 Hz, 1H), 8.13 (s, 1H), 7.95 (d, *J* = 8.2 Hz, 1H), 7.58–7.49 (m, 2H), 7.43 (d, *J* = 5.1 Hz, 1H), 7.24 (d, *J* = 8.3 Hz, 1H), 4.00 (s, 3H), 3.46 (q, *J* = 6.8 Hz, 2H), 3.27 (q, *J* = 6.7 Hz, 2H), 2.24 (s, 3H), 1.20 (t, *J* = 7.0 Hz, 3H), 1.09 (t, *J* = 6.9 Hz, 3H); ^13^C NMR (100 MHz, DMSO-*d*_6_) *δ* 186.0, 166.8, 165.4, 161.6, 161.2, 159.5, 151.4, 148.2, 141.9, 137.9, 137.3, 137.1, 134.4, 132.2, 130.2, 130.1, 127.7, 127.6, 123.8, 122.3, 120.6, 117.3, 116.8, 111.9, 111.2, 107.5, 41.7, 38.2, 33.6, 17.7, 14.2, 12.8; HRMS (*m/z*): [M+H]^+^ calcd for C_29_H_25_N_7_O_3_: 562.2561, found: 562.2566.

#### 1-Methyl-*N*-(4-methyl-3-((4-(pyridin-3-yl)pyrimidin-2-yl)amino)phenyl)-3-(2-(4-methylpiperazin-1-yl)-2-oxoacetyl)-1*H*-indole-6-carboxamide (I8)

White solid, 64.5 mg, yield: 36.1%, Mp: 235.1–237.0 °C; ^1^H NMR (400 MHz, DMSO-*d*_6_) *δ* 10.25 (s, 1H), 9.29 (d, *J* = 1.9 Hz, 1H), 8.98 (s, 1H), 8.68 (dd, *J* = 4.7, 1.4 Hz, 1H), 8.52 (d, *J* = 5.1 Hz, 1H), 8.48 (dt, *J* = 8.0, 1.9 Hz, 1H), 8.38 (s, 1H), 8.28 (s, 1H), 8.21 (d, *J* = 8.3 Hz, 1H), 8.12 (d, *J* = 1.9 Hz, 1H), 7.96 (dd, *J* = 8.3, 1.3 Hz, 1H), 7.55–7.50 (m, 2H), 7.43 (d, *J* = 5.1 Hz, 1H), 7.24 (d, *J* = 8.4 Hz, 1H), 4.00 (s, 3H), 3.64 (t, *J* = 4.7 Hz, 2H), 3.35 (t, *J* = 4.8 Hz, 2H), 2.45 (t, *J* = 4.8 Hz, 2H), 2.30 (t, *J* = 4.7 Hz, 2H), 2.24 (s, 3H), 2.22 (s, 3H); ^13^C NMR (100 MHz, DMSO-*d*_6_) *δ* 185.6, 165.5, 165.3, 163.1, 161.6, 161.2, 159.5, 151.4, 148.2, 142.3, 137.9, 137.2, 134.4, 132.2, 130.3, 130.1, 127.6, 123.8, 122.4, 120.6, 117.3, 116.9, 112.0, 111.2, 107.5, 99.5, 54.5, 53.9, 45.5, 33.6, 17.7; HRMS (*m/z*): [M+H]^+^ calcd for C_29_H_25_N_7_O_3_: 589.2670, found: 589.2666.

#### 1-Benzyl-3-(2-(diethylamino)-2-oxoacetyl)-*N*-(4-methyl-3-((4-(pyridin-3-yl)pyrimidin-2-yl)amino)phenyl)-1*H-*indole-6-carboxamide (I9)

White solid, 93.3 mg, yield: 55.4%, Mp: 216.0–218.0 °C; ^1^H NMR (400 MHz, DMSO-*d*_6_) *δ* 10.21 (s, 1H), 9.29 (s, 1H), 8.97 (s, 1H), 8.68 (dd, *J* = 4.8, 1.6 Hz, 1H), 8.51 (d, *J* = 5.1 Hz, 1H), 8.50–8.46 (m, 2H), 8.25 (s, 1H), 8.22 (d, *J* = 8.4 Hz, 1H), 8.11 (s, 1H), 7.96 (d, *J* = 8.4 Hz, 1H), 7.51–7.47 (m, 2H), 7.43 (d, *J* = 5.2 Hz, 1H), 7.37–7.32 (m, 2H), 7.29 (d, *J* = 6.7 Hz, 3H), 7.22 (d, *J* = 8.5 Hz, 1H), 5.67 (s, 2H), 3.46 (q, *J* = 7.1 Hz, 2H), 3.27 (q, *J* = 7.0 Hz, 2H), 2.24 (s, 3H), 1.19 (t, *J* = 7.1 Hz, 3H), 1.08 (t, *J* = 7.0 Hz, 3H); ^13^C NMR (100 MHz, DMSO-*d*_6_) *δ* 186.2, 166.7, 165.2, 161.6, 161.2, 159.5, 151.4, 148.2, 141.3, 137.8, 137.1, 136.5, 134.4, 132.2, 130.5, 130.1, 128.8, 127.9, 127.9, 127.6, 127.2, 123.8, 122.3, 120.7, 117.4, 116.9, 112.5, 111.6, 107.5, 99.5, 49.8, 41.7, 38.2, 17.7, 14.1, 12.7; HRMS (*m/z*): [M+H]^+^ calcd for C_29_H_25_N_7_O_3_: 638.2874, found: 638.2878.

#### 3-(2-(Dimethylamino)-2-oxoacetyl)-1-(4-fluorobenzyl)-*N*-(4-methyl-3-((4-(pyridin-3-yl)pyrimidin-2-yl)amino) phenyl)-1*H*-indole-6-carboxamide (I10)

White solid, 78.2 mg, yield: 45.9%, Mp: 220.4–222.5 °C; ^1^H NMR (400 MHz, DMSO-*d*_6_) *δ* 10.49 (s, 1H), 9.28 (d, *J* = 1.6 Hz, 1H), 8.99 (s, 1H), 8.67 (dd, *J* = 4.8, 1.6 Hz, 1H), 8.58 (s, 1H), 8.56 (s, 1H), 8.50 (d, *J* = 5.2 Hz, 1H), 8.48 (dt, *J* = 8.0, 2.0 Hz, 1H), 8.20 (d, *J* = 8.4 Hz, 1H), 8.17 (d, *J* = 2.1 Hz, 1H), 7.98 (dd, *J* = 8.4, 1.4 Hz, 1H), 7.61 (dd, *J* = 8.2, 2.2 Hz, 1H), 7.49 (dd, *J* = 8.0, 4.8 Hz, 1H), 7.47–7.43 (m, 2H), 7.42 (d, *J* = 5.2 Hz, 1H), 7.19 (q, *J* = 8.8 Hz, 3H), 5.70 (s, 2H), 3.02 (s, 3H), 2.95 (s, 3H), 2.23 (s, 3H); ^13^C NMR (100 MHz, DMSO-*d*_6_) *δ* 186.2, 166.9, 165.2, 161.6, 161.2, 160.5, 159.5, 151.4, 148.2, 141.5, 137.7, 137.3, 136.4, 134.5, 132.9, 132.2, 130.3, 129.9, 129.7, 129.6, 127.9, 127.7, 123.8, 122.6, 120.7, 117.6, 117.1, 115.7, 115.5, 112.5, 111.8, 107.5, 49.1, 36.9, 33.6, 17.7; HRMS (*m/z*): [M+H]^+^ calcd for C_29_H_25_N_7_O_3_: 628.2467, found: 628.2463.

#### 1-(4-fluorobenzyl)-*N*-(4-methyl-3-((4-(pyridin-3-yl)pyrimidin-2-yl)amino)phenyl)-3-(2-oxo-2-(piperidin-1-yl) acetyl)-1*H*-indole-6-carboxamide (I11)

Yellow solid, 77.3 mg, yield: 47.3%, Mp: 229.4–231.5 °C; ^1^H NMR (400 MHz, DMSO-*d*_6_) *δ* 10.25 (s, 1H), 9.29 (d, *J* = 1.5 Hz, 1H), 8.98 (s, 1H), 8.67 (dd, *J* = 4.8, 1.6 Hz, 1H), 8.53 (s, 1H), 8.51 (d, *J* = 5.1 Hz, 1H), 8.50–8.46 (m, 1H), 8.32 (s, 1H), 8.22 (d, *J* = 8.4 Hz, 1H), 8.11 (d, *J* = 2.1 Hz, 1H), 7.96 (dd, *J* = 8.4, 1.4 Hz, 1H), 7.53–7.48 (m, 2H), 7.44–7.39 (m, 3H), 7.20 (q, *J* = 9.0 Hz, 3H), 5.66 (s, 2H), 3.59 (s, 2H), 3.30 (t, *J* = 5.4 Hz, 2H), 2.24 (s, 3H), 1.61 (s, 4H), 1.40 (s, 2H); ^13^C NMR (100 MHz, DMSO-*d*_6_) *δ* 186.3, 165.3, 165.2, 161.6, 161.2, 160.5, 159.5, 151.4, 148.2, 141.3, 137.8, 137.2, 136.4, 134.4, 132.8, 132.2, 130.5, 130.0, 129.6, 129.5, 127.8, 127.6, 123.8, 122.4, 120.8, 117.4, 116.9, 115.8, 115.6, 112.7, 111.5, 107.5, 49.1, 46.5, 41.4, 25.8, 25.1, 23.9, 17.7; HRMS (*m/z*): [M+H]^+^ calcd for C_39_H_34_FN_7_O_3_: 668.2780, found: 668.2777.

#### 3-(2-(Diethylamino)-2-oxoacetyl)-1-(4-fluorobenzyl)-*N*-(4-methyl-3-((4-(pyridin-3-yl)pyrimidin-2-yl)amino) phenyl)-1*H*-indole-6-carboxamide (I12)

Yellow solid, 88.8 mg, yield: 53.7%, Mp: 233.4–235.5 °C; ^1^H NMR (400 MHz, DMSO-*d*_6_) *δ* 10.48 (s, 1H), 9.28 (s, 1H), 8.99 (s, 1H), 8.67 (dd, *J* = 4.8, 1.6 Hz, 1H), 8.56 (s, 1H), 8.52–8.46 (m, 3H), 8.22–8.16 (m, 2H), 7.99–7.92 (m, 1H), 7.61 (dd, *J* = 8.2, 2.1 Hz, 1H), 7.51–7.41 (m, 4H), 7.19 (q, *J* = 8.8 Hz, 3H), 5.71 (s, 2H), 3.51–3.41 (m, 2H), 3.26 (q, *J* = 7.0 Hz, 2H), 2.23 (s, 3H), 1.19 (t, *J* = 7.1 Hz, 3H), 1.07 (t, *J* = 7.0 Hz, 3H); ^13^C NMR (100 MHz, DMSO-*d*_6_) *δ* 186.2, 166.7, 165.2, 161.6, 161.2, 160.5, 159.5, 151.3, 148.2, 141.1, 137.7, 137.3, 136.4, 134.4, 132.9, 132.2, 130.3, 129.9, 129.7, 129.6, 127.8, 127.7, 123.8, 122.6, 120.7, 117.6, 117.1, 115.7, 115.5, 112.5, 111.8, 107.5, 49.0, 41.7, 38.2, 17.7, 14.1, 12.7; HRMS (*m/z*): [M+H]^+^ calcd for C_38_H_34_FN_7_O_3_: 656.2780, found: 656.2784.

#### 1-(4-Chlorobenzyl)-3-(2-(dimethylamino)-2-oxoacetyl)-*N*-(4-methyl-3-((4-(pyridin-3-yl)pyrimidin-2-yl)amino) phenyl)-1*H*-indole-6-carboxamide (I13)

White solid, 78.0 mg, yield: 46.6%, Mp: 227.5–229.4 °C; ^1^H NMR (400 MHz, DMSO-*d*_6_) *δ* 10.21 (s, 1H), 9.29 (s, 1H), 8.97 (s, 1H), 8.67 (dd, *J* = 4.8, 1.6 Hz, 1H), 8.56 (s, 1H), 8.51 (d, *J* = 5.2 Hz, 1H), 8.48 (dt, *J* = 8.0, 1.7 Hz, 1H), 8.24–8.21 (m, 2H), 8.09 (d, *J* = 2.0 Hz, 1H), 7.96 (d, *J* = 9.8 Hz, 1H), 7.51–7.47 (m, 2H), 7.44–7.40 (m, 3H), 7.32 (d, *J* = 8.6 Hz, 2H), 7.22 (d, *J* = 8.6 Hz, 1H), 5.66 (s, 2H), 3.02 (s, 3H), 2.96 (s, 3H), 2.23 (s, 3H); ^13^C NMR (100 MHz, DMSO-*d*_6_) *δ* 186.2, 166.9, 165.2, 161.6, 161.2, 159.5, 151.4, 148.2, 141.7, 137.8, 137.1, 136.4, 135.6, 134.4, 132.6, 132.2, 130.6, 130.1, 129.1, 128.8, 127.9, 127.6, 123.8, 122.4, 120.8, 117.4, 116.9, 112.6, 111.5, 107.5, 49.1, 36.9, 33.6, 17.7; HRMS (*m/z*): [M+H]^+^ calcd for C_36_H_30_ClN_7_O_3_: 644.2171, found: 644.2175.

#### 1-(4-Chlorobenzyl)-3-(2-(diethylamino)-2-oxoacetyl)-*N*-(4-methyl-3-((4-(pyridin-3-yl)pyrimidin-2-yl)amino) phenyl)-1*H*-indole-6-carboxamide (I14)

Off-white solid, 87.1 mg, yield: 53.5%, Mp: 231.1–233.0 °C; ^1^H NMR (400 MHz, DMSO-*d*_6_) *δ* 10.20 (s, 1H), 9.28 (d, *J* = 1.8 Hz, 1H), 8.97 (s, 1H), 8.67 (dd, *J* = 4.7, 1.5 Hz, 1H), 8.51 (d, *J* = 5.1 Hz, 1H), 8.50–8.46 (m, 2H), 8.21 (d, *J* = 8.7 Hz, 2H), 8.09 (d, *J* = 2.0 Hz, 1H), 7.95 (dd, *J* = 8.4, 1.4 Hz, 1H), 7.52–7.47 (m, 2H), 7.44–7.40 (m, 3H), 7.31 (d, *J* = 8.5 Hz, 2H), 7.22 (d, *J* = 8.6 Hz, 1H), 5.67 (s, 2H), 3.46 (q, *J* = 7.1 Hz, 2H), 3.27 (q, *J* = 7.1 Hz, 2H), 2.23 (s, 3H), 1.19 (t, *J* = 7.1 Hz, 3H), 1.08 (t, *J* = 7.0 Hz, 3H); ^13^C NMR (100 MHz, DMSO-*d*_6_) *δ* 186.2, 166.7, 165.2, 161.6, 161.2, 159.5, 151.4, 148.2, 141.3, 137.8, 137.1, 136.4, 135.6, 134.4, 132.6, 132.2, 130.6, 130.0, 129.1, 128.8, 127.9, 127.6, 123.8, 122.3, 120.8, 117.4, 116.9, 112.6, 111.5, 107.5, 49.1, 41.7, 38.2, 17.7, 14.1, 12.7; HRMS (*m/z*): [M+H]^+^ calcd for C_38_H_34_ClN_7_O_3_: 672.2484, found: 672.2481.

#### 1-(4-Chlorobenzyl)-*N*-(4-methyl-3-((4-(pyridin-3-yl)pyrimidin-2-yl)amino)phenyl)-3-(2-(4-methylpiperazin-1-yl)-2-oxoacetyl)-1*H*-indole-6-carboxamide (I15)

Yellow solid, 86.4 mg, yield: 54%, Mp; 229.6–231.6 °C; ^1^H NMR (400 MHz, DMSO-*d*_6_) *δ* 10.22 (s, 1H), 9.29 (s, 1H), 8.98 (s, 1H), 8.68 (d, *J* = 3.5 Hz, 1H), 8.62 (s, 1H), 8.51 (d, *J* = 5.1 Hz, 1H), 8.48 (dt, *J* = 8.0, 1.9 Hz, 1H), 8.25 (t, *J* = 4.1 Hz, 2H), 8.09 (s, 1H), 7.98 (d, *J* = 8.5 Hz, 1H), 7.51–7.47 (m, 2H), 7.44–7.41 (m, 3H), 7.34 (d, *J* = 8.5 Hz, 2H), 7.23 (d, *J* = 8.6 Hz, 1H), 5.67 (s, 2H), 3.10 (s, 2H), 2.94 (s, 2H), 2.67 (s, 3H), 2.24 (s, 3H); ^13^C NMR (100 MHz, DMSO-*d*_6_) *δ* 184.9, 165.2, 165.1, 161.6, 161.2, 159.5, 151.4, 148.2, 142.1, 137.9, 137.1, 136.5, 135.5, 134.5, 132.6, 132.2, 130.7, 130.1, 129.1, 128.9, 127.9, 127.7, 123.8, 122.6, 120.9, 117.4, 117.0, 112.6, 111.5, 107.6, 53.1, 52.6, 52.6, 49.2, 17.7; HRMS (*m/z*): [M+H]^+^ calcd for C_39_H_35_ClN_8_O_3_: 699.2593, found: 699.2588.

### 3.3. Molecular docking

Molecular docking was carried out using the Surflex-Dock module in Sybyl 2.1.1 software package. The crystal structure of Bcr-Abl kinase (PDB code: 1IEP) was download from RCSB Protein Data Bank. A series of protein treatments are needed before the formal docking. Firstly, the initial cocrystalized ligand and water molecules were removed from the crystal structure, hydrogens were added and side chains were fixed during protein preparation. The protein was prepared by using biopolymer module. Then Tripos force field was used to minimize the protein structure and partial atomic charges were calculated by the Gasteiger-Huckel method. All the steps parameters were set to the default value of 100 in the window of staged minimization. Subsequently, the protein was prepared using biopolymer module implemented in Sybyl. Protein structure minimization was performed by applying the Tripos force field and partial atomic charges were calculated by the Gasteiger-Huckel method. The gradient of termination was defined as 0.005 kcal/(mol*A) and the maximum number of interactions were 10,000.

## Table of contents

**Table:** 

1. The general synthetic procedure for intermediates **4** and **5**	S2–S3
2. ^1^H and ^13^C NMR spectra of compounds	S4–S32

### 1. Experimental procedures and physical data of compounds1.1 General procedure for the preparation of compound 4

To a mixture of NaH (0.44 g,18.0 mmol) in *N,N*-dimethylformamide (7.0 mL), was added a solution of methyl 1*H*-indole-6-carboxylate (1.75 g, 10.0 mmol) in DMF (3.0 mL) at 0 °C. The reaction mixture was allowed to stir at room temperature for 2 h. Then 1-chloro-4-(chloromethyl)benzene (1.76 g, 11 mmol) was added to the mixture and it was allowed to stir at 40 °C for 6 h. The reaction mixture was cooled to room temperature, and diluted with water. The aqueous layer was extracted with ethyl acetate twice. The organic layer was combined, dried, filtered, and concentrated to afford the title compound, and used in the next step without further purification.

***Methyl 1-methyl-1H-indole-6-carboxylate* (4a)**. Brown solid, yield: 89%.

***Methyl 1-(4-fluorobenzyl)-1H-indole-6-carboxylate* (4b)**. White solid, yield: 85.7%.

***Methyl 1-(4-chlorobenzyl)-1H-indole-6-carboxylate* (4c)**. White solid, yield: 89%. ^1^H NMR (400 MHz, CDCl_3_) δ 7.99–7.98 (m, 1H), 7.76 (dd, *J* = 8.4, 1.4 Hz, 1H), 7.60 (dd, *J* = 8.4, 0.7 Hz, 1H), 7.22–7.21 (m, 1H), 7.20-7.18 (m, 2H), 6.96 (d, *J* = 8.7 Hz, 2H), 6.54 (dd, *J* = 3.1, 0.9 Hz, 1H), 5.27 (s, 2H), 3.85 (s, 3H).

***Methyl 1-benzyl-1H-indole-6-carboxylate* (4d)**. Yellow solid, yield: 84.3%.

#### 1.2 General procedure for the preparation of compound 5

To a solution of methyl 1*H*-indole-6-carboxylate (3.0 g, 17.1 mmol) in 30 mL of ethyl ether, was added oxalyl chloride (2.9 mL, 34.3 mmol) slowly by an addition funnel at 0 °C. The reaction mixture was allowed to stir at room temperature for 2 h. The precipitate was collected by filtration, and used in the next step as obtain.

To a solution of dimethylamine hydrochloride (1.69 g, 20.7 mmol) in 50 mL of DCM, was added triethylamine (5.71 g, 56.5 mmol) at 0 °C. Then the above made methyl 3-(2-chloro-2-oxoacetyl)-1*H*-indole-6-carboxylate (5.0 g, 18.8 mmol) was added by small portions. After addition, the reaction mixture was allowed to stir at room temperature for 5 h. The reaction mixture was diluted with water, and the organic layer was isolated, washed with brine, dried with sodium sulfate, filtered, and concentrated. The residue was title compound and usually used in next step without further purification.

***Methyl 3-(2-(dimethylamino)-2-oxoacetyl)-1H-indole-6-carboxylate* (5a)**. Yellow solid, yield: 96.1%. ^1^H NMR (400 MHz, CD_3_OD) δ: 8.26 (d, *J* = 8.4 Hz, 1H), 8.21–8.20 (m 1H), 8.19 (s, 1H), 7.94 (dd, *J* = 8.4, 1.5 Hz, 1H), 3.94 (s, 3H), 3.11 (s, 3H), 3.04 (s, 3H).

***Methyl 3-(2-(diethylamino)-2-oxoacetyl)-1H-indole-6-carboxylate* (5b)**. Yellow solid, yield: 64.2%. Used in next step as obtained.

***Methyl 3-(2-(isopropyl(methyl)amino)-2-oxoacetyl)-1H-indole-6-carboxylate* (5c)**. Yellow solid, yield: 59.8%. Used in next step as obtained.

***Methyl 3-(2-oxo-2-(piperidin-1-yl)acetyl)-1H-indole-6-carboxylate* (5d)**. Yellow solid, yield: 71.3%. Used in next step as obtained.

***Methyl 3-(2-morpholino-2-oxoacetyl)-1H-indole-6-carboxylate* (5e)**. Yellow solid, yield: 67.2%. Used in next step as obtained.

***Methyl 3-(2-(4-methyliperazin-1-yl)-2-oxoacetyl)-1H-indole-6-carboxylate* (5f)**. White solid, yield: 68.4%. ^1^H NMR (400 MHz, CDCl_3_) δ: 10.08 (s, 1H), 8.34 (d, *J* = 8.4 Hz, 1H), 8.13 (s, 1H), 8.02–7.95 (m, 2H), 3.94 (s, 3H), 3.79 (t, *J* = 4.8 Hz, 2H), 3.55 (t, *J* = 5.0 Hz, 2H), 2.52 (t, *J* = 5.1 Hz, 2H), 2.43 (t, *J* = 4.9 Hz, 2H), 2.33 (s, 3H).

***Methyl 3-(2-(diethylamino)-2-oxoacetyl)-1-methyl-1H-indole-6-carboxylate* (5g)**. White solid, yield: 68.2%. Used in next step as obtained.

***Methyl 1-methyl-3-(2-(4-methylpiperazin-1-yl)-2-oxoacetyl)-1H-indole-6-carboxylate* (5h)**. Pale yellow solid, yield: 64.8%. Used in next step as obtained.

***Methyl 1-benzyl-3-(2-(diethylamino)-2-oxoacetyl)-1H-indole-6-carboxylate* (5i)**. Pale yellow solid, yield: 85.3%. Used in next step as obtained.

***Methyl 3-(2-(dimethylamino)-2-oxoacetyl)-1-(4-fluorobenzyl)-1H-indole-6-carboxyl-ate* (5j)**. Pale yellow solid, yield: 61.3 %. Used in next step as obtained.

***Methyl 1-(4-fluorobenzyl)-3-(2-oxo-2-(piperidin-1-yl)acetyl)-1H-indole-6-carboxyl-ate* (5k)**. Pale yellow solid, yield: 58.8%. Used in next step as obtained.

***Methyl 3-(2-(diethylamino)-2-oxoacetyl)-1-(4-fluorobenzyl)-1H-indole-6-carboxyl-ate* (5l)**. Pale yellow solid, yield: 69.9%. Used in next step as obtained.

***Methyl 3-(2-(dimethylamino)-2-oxoacetyl)-1-(4-chlorobenzyl)-1H-indole-6-carboxyl-ate* (5m)**. Pale yellow solid, yield: 84.3%. Used in next step as obtained.

***Methyl 3-(2-(diethylamino)-2-oxoacetyl)-1-(4-chlorobenzyl)-1H-indole-6-carboxyl-ate* (5n)**. Pale yellow solid, yield: 73.5%. Used in next step as obtained.

***Methyl 1-(4-chlorobenzyl)-3-(2-(4-methylpiperazin-1-yl)-2-oxoacetyl)-1H-indole-6-carboxylate* (5o)**. Pale yellow solid, yield: 71.1%. ^1^H NMR (400 MHz, CDCl_3_) δ 8.38 (d, *J* = 8.4 Hz, 1H), 8.04 (s, 2H), 8.01 (d, *J* = 8.4 Hz, 1H), 7.30 (d, *J* = 8.6 Hz, 2H), 7.10 (d, *J* = 8.7 Hz, 2H), 5.36 (s, 2H), 3.92 (s, 3H), 3.75 (t, *J* = 5.0 Hz, 2H), 3.55 (t, *J* = 5.0 Hz, 2H), 2.48 (t, *J* = 5.1 Hz, 2H), 2.39 (t, *J* = 5.0 Hz, 2H), 2.31 (s, 3H).

### 2. ^1^H and ^13^C NMR spectra of compounds

Figure S1 ^1^H-NMR spectrum of **I1**.

Figure S2 ^13^C-NMR spectrum of **I1**.

Figure S3 ^1^H-NMR spectrum of **I2**.

Figure S4 ^13^C-NMR spectrum of **I2**.

Figure S5 ^1^H-NMR spectrum of **I3**.

Figure S6 ^13^C-NMR spectrum of **I3**.

Figure S7 ^1^H-NMR spectrum of **I4**.

Figure S8 ^13^C-NMR spectrum of **I4**.

Figure S9 ^1^H-NMR spectrum of **I5**.

Figure S10 ^13^C-NMR spectrum of **I5**.

Figure S11 ^1^H-NMR spectrum of **I6**.

Figure S12 ^13^C-NMR spectrum of **I6**.

Figure S13 ^1^H-NMR spectrum of **I7**.

Figure S14 ^13^C-NMR spectrum of **I7**.

Figure S15 ^1^H-NMR spectrum of **I8**.

Figure S16 ^13^C-NMR spectrum of **I8**.

Figure S17 ^1^H-NMR spectrum of **I9**.

Figure S18 ^13^C-NMR spectrum of **I9**.

Figure S19 ^1^H-NMR spectrum of **I10**.

Figure S20 ^13^C-NMR spectrum of **I10**.

Figure S21 ^1^H-NMR spectrum of **I11**.

Figure S22 ^13^C-NMR spectrum of **I11**.

Figure S23 ^1^H-NMR spectrum of **I12**.

Figure S24 ^13^C-NMR spectrum of **I12**.

Figure S25 ^1^H-NMR spectrum of **I13**.

Figure S26 ^13^C-NMR spectrum of **I13**.

Figure S27 ^1^H-NMR spectrum of **I14**.

Figure S28 ^13^C-NMR spectrum of **I14**.

Figure S29 ^1^H-NMR spectrum of **I15**.

Figure S30 ^13^C-NMR spectrum of **I15**.

## Figures and Tables

**Table:** Structures and antitumor activities of compounds **I1**–**I15**.

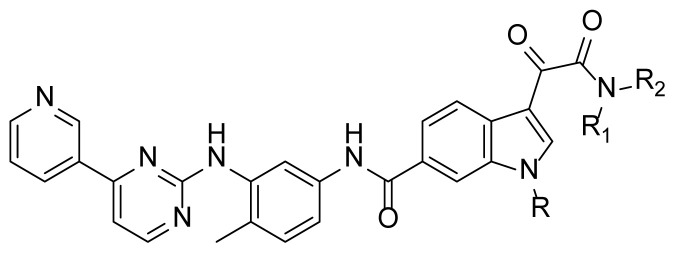				
Compd.	R	NR_1_R_2_	IC_50_ (μM)	
			K562	K562R
			4.1 ± 2.3	7.6 ± 3.4
**I2**	H	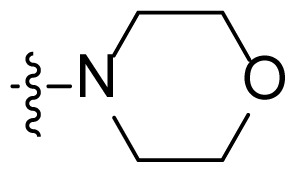	0.8 ± 0.06	0.7 ± 0.1
**I3**	H	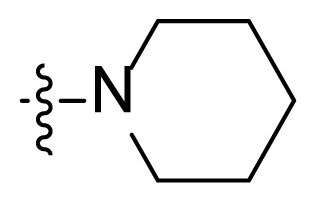	2.8 ± 0.7	2.4 ± 0.6
**I4**	H	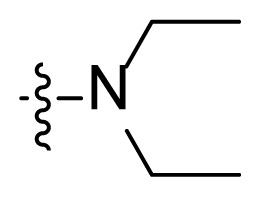	5.6 ± 1.3	5.8 ± 2.1
**I5**	H	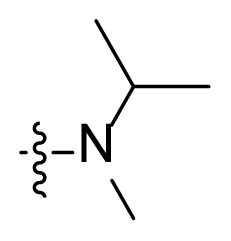	6.1 ± 1.1	7.8 ± 1.6
**I6**	H	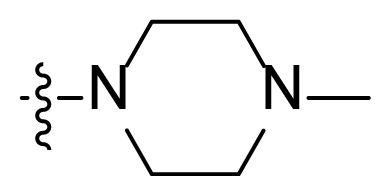	5.2 ± 0.8	4.0 ± 1.2
**I7**	Me	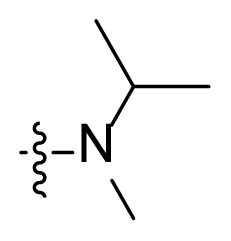	13.2 ± 4.1	13.6 ± 2.2
**I8**	Me	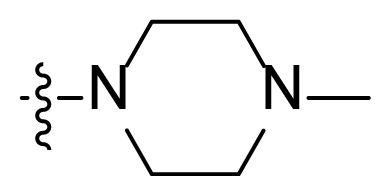	16.1 ± 2.1	12.4 ± 3.3
**I9**	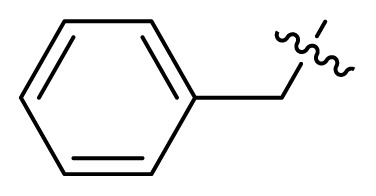	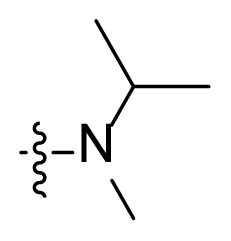	10.6 ± 3.4	13.2 ± 1.4
**I10**	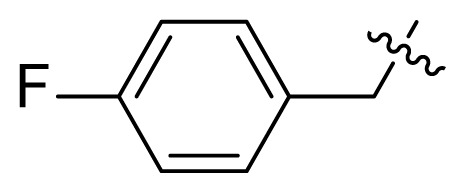	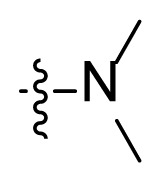	9.7 ± 1.4	14.7 ± 5.2
**I11**	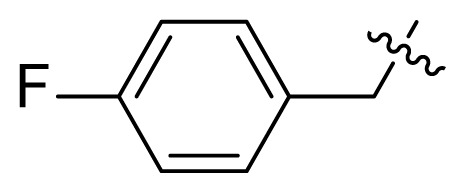	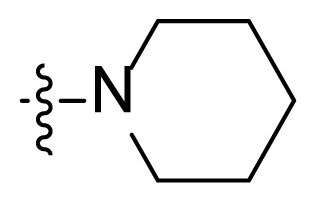	18.2 ± 1.1	18.6 ± 2.2
**I12**	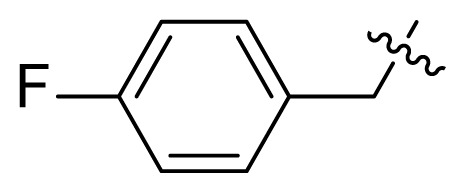	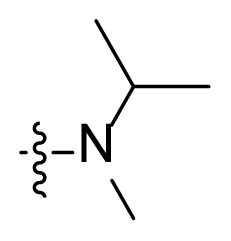	16.5 ± 1.7	18.5 ± 1.1
**I13**	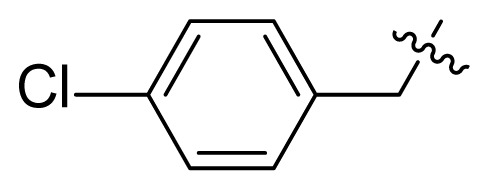	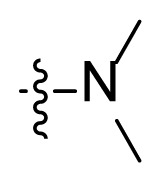	17.8 ± 2.1	15.9 ± 1.3
**I14**	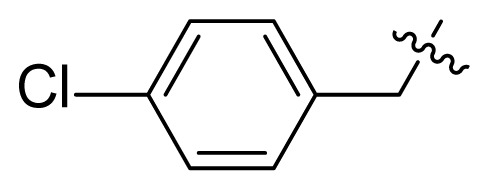	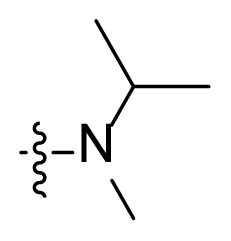	12.6 ± 4.4	15.1 ± 3.7
**I15**	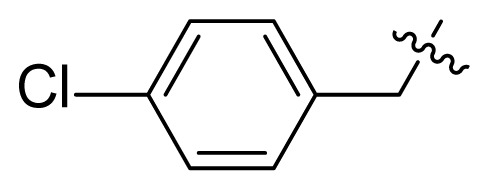	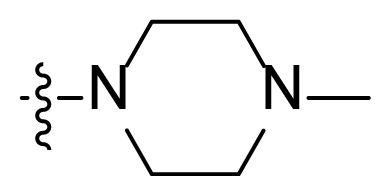	22.5 ± 0.8	22.8 ± 0.7
**Imatinib**	/	/	3.8 ± 0.9	78.3 ± 7.6
