# Supplementary figures and images for: Privileged fragment-based design, synthesis and in vitro antitumor activity of imatinib analogues
Source: Turk J Chem. 2023 Feb 14;47(2):426–35. doi: 10.55730/1300-0527.3549 (PMC10388080; doi:10.55730/1300-0527.3549)

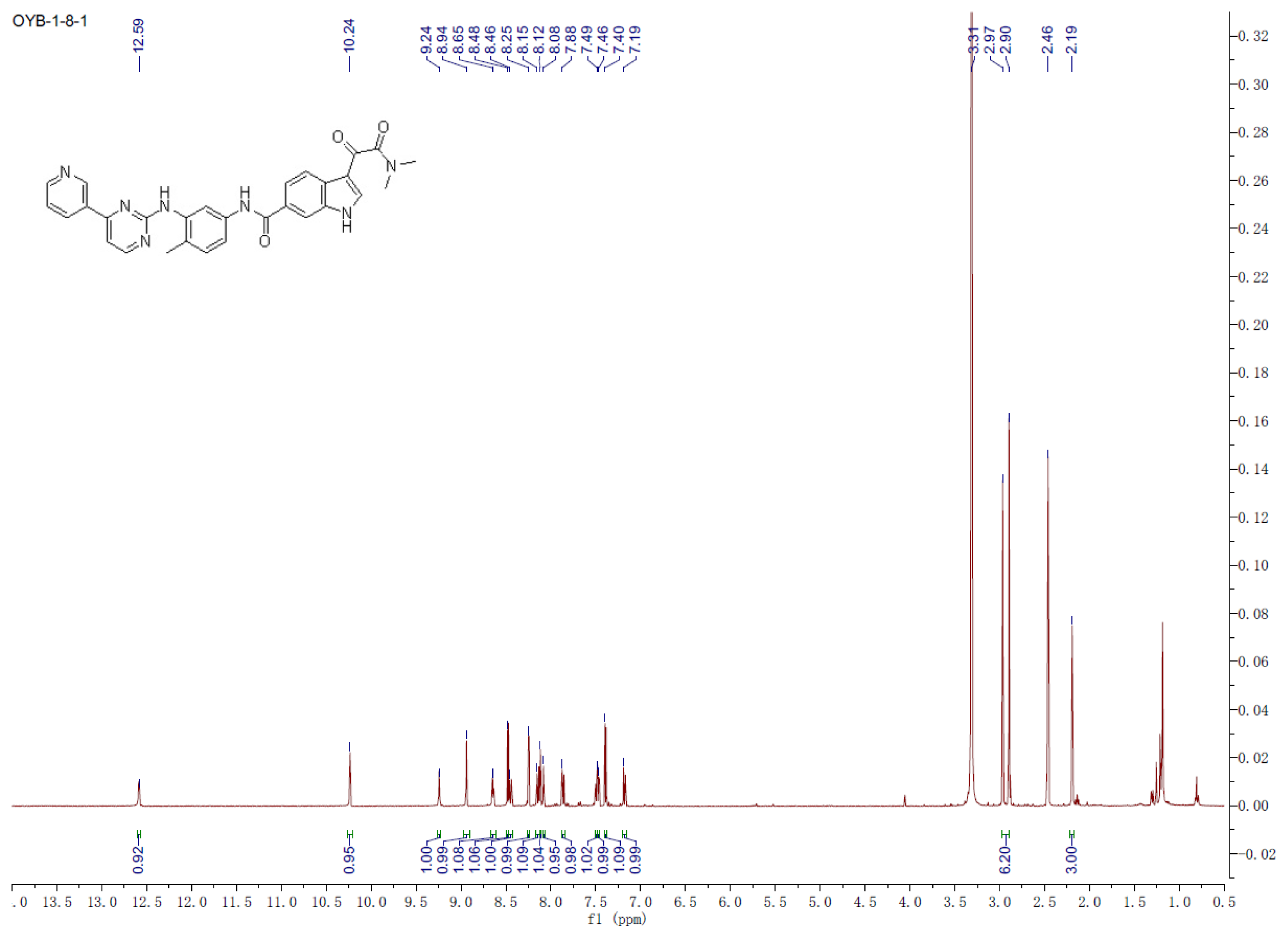

Supplement: Figure S1 — 1H-NMR spectrum of I1. [file turkjchem-47-2-426s1.tif]

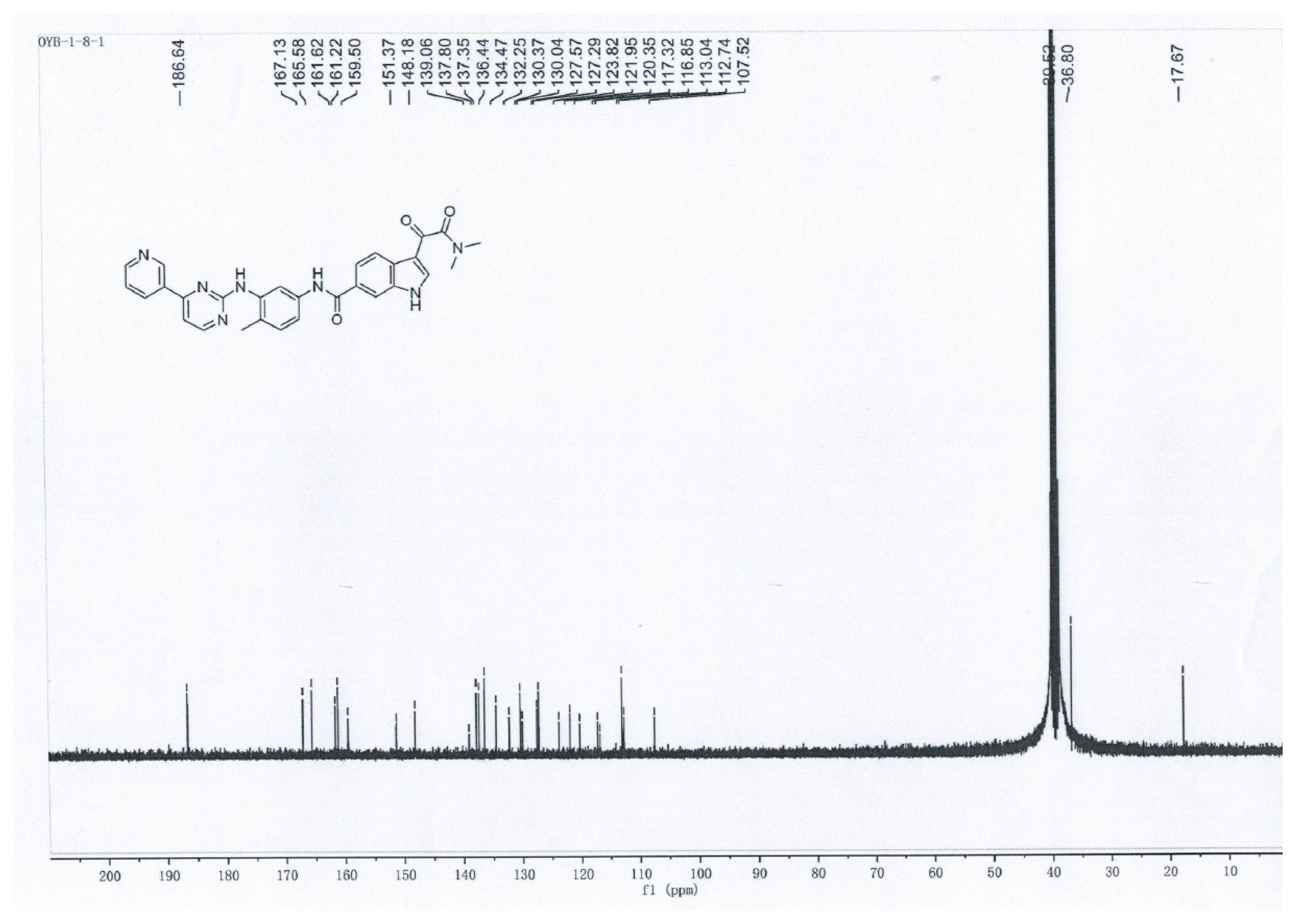

Supplement: Figure S2 — 13C-NMR spectrum of I1. [file turkjchem-47-2-426s2.tif]

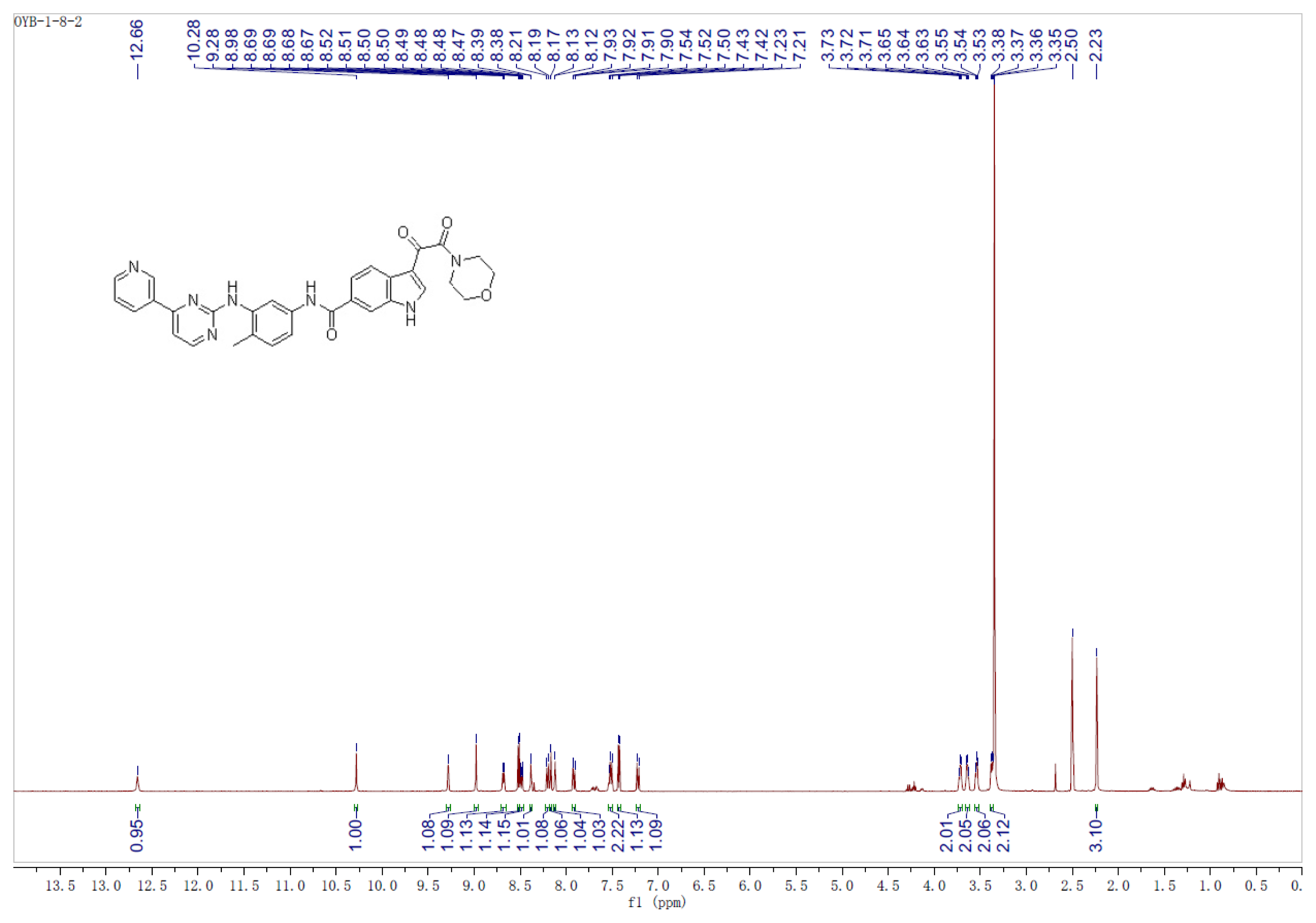

Supplement: Figure S3 — 1H-NMR spectrum of I2. [file turkjchem-47-2-426s3.tif]

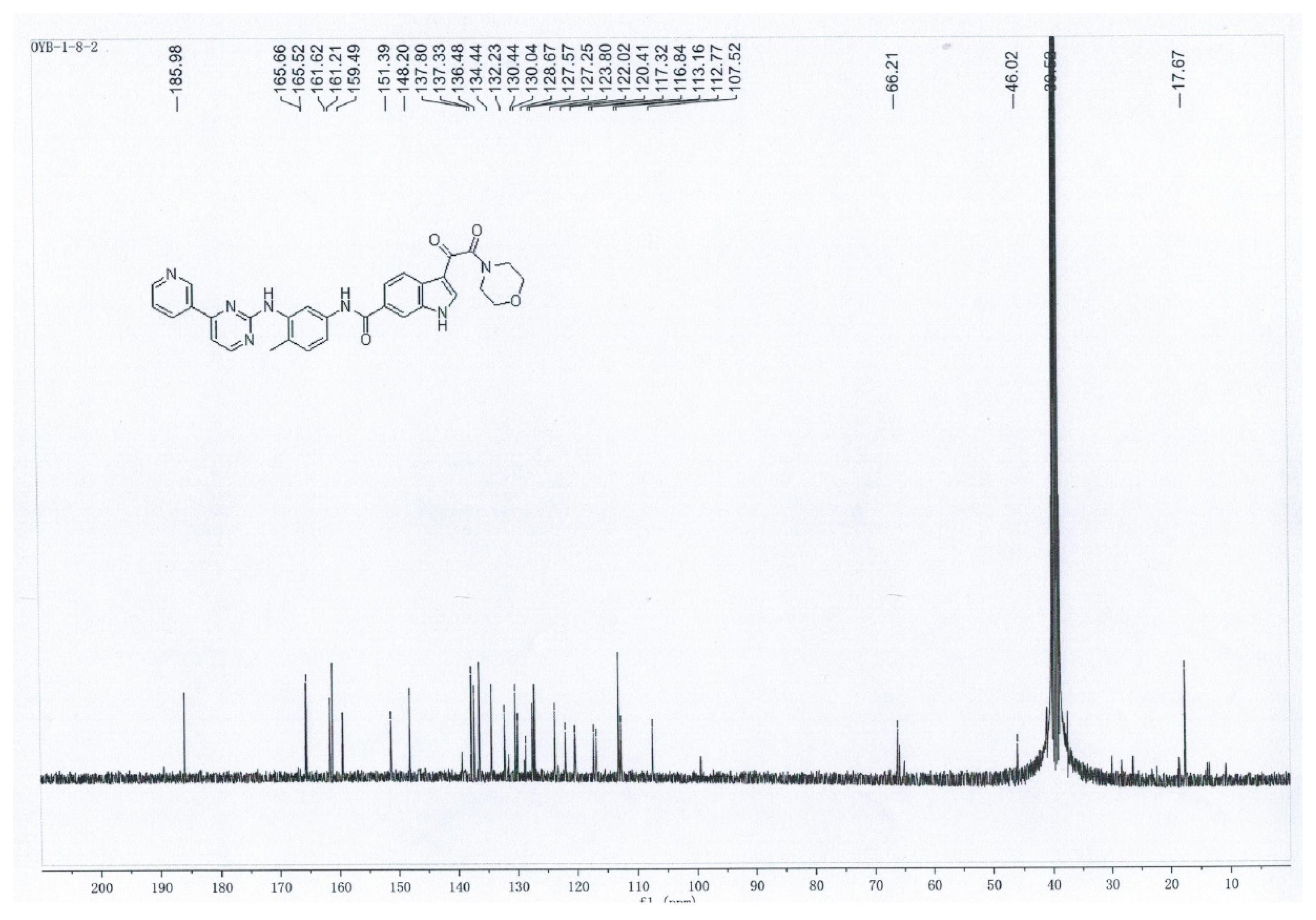

Supplement: Figure S4 — 13C-NMR spectrum of I2. [file turkjchem-47-2-426s4.tif]

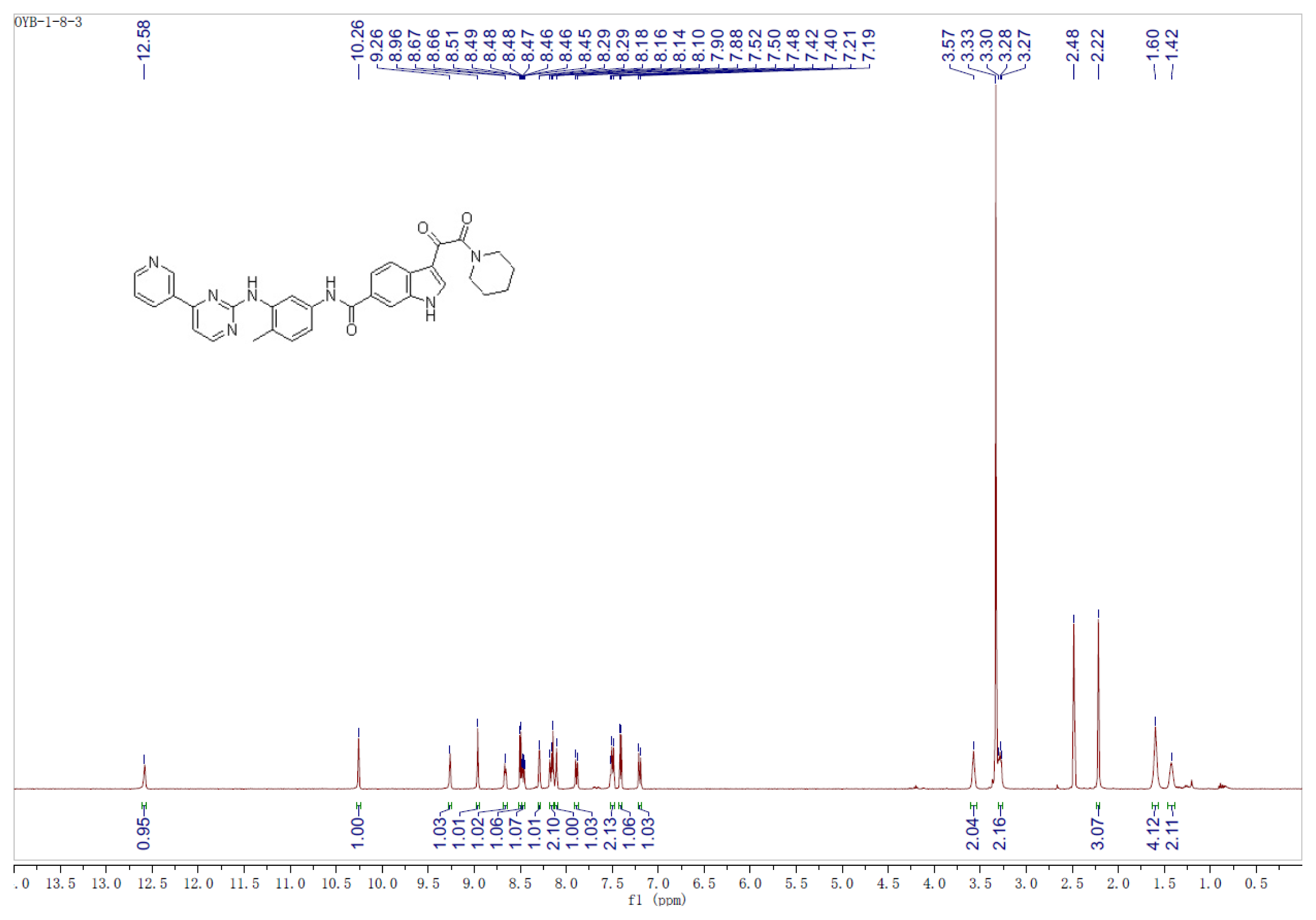

Supplement: Figure S5 — 1H-NMR spectrum of I3. [file turkjchem-47-2-426s5.tif]

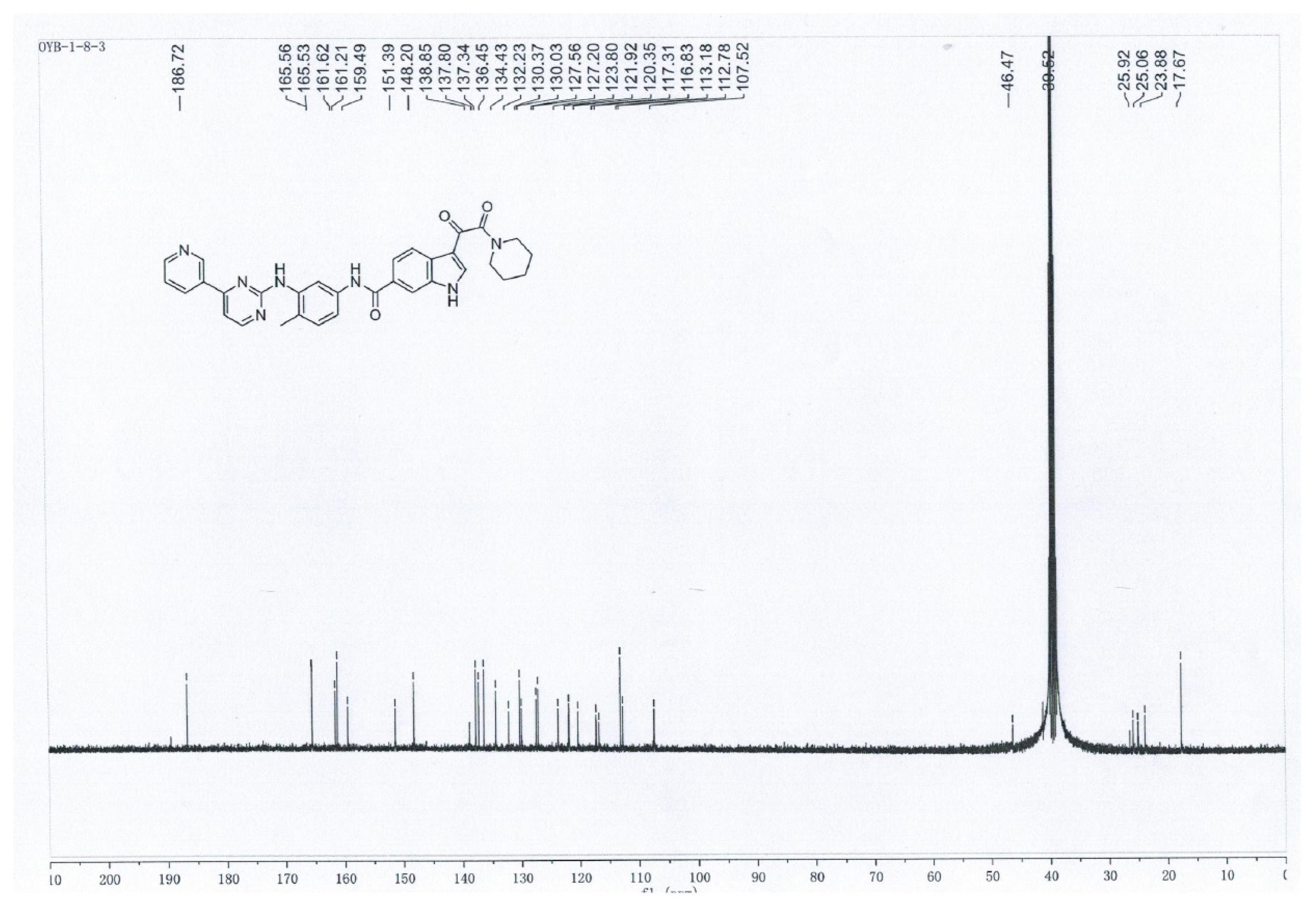

Supplement: Figure S6 — 13C-NMR spectrum of I3. [file turkjchem-47-2-426s6.tif]

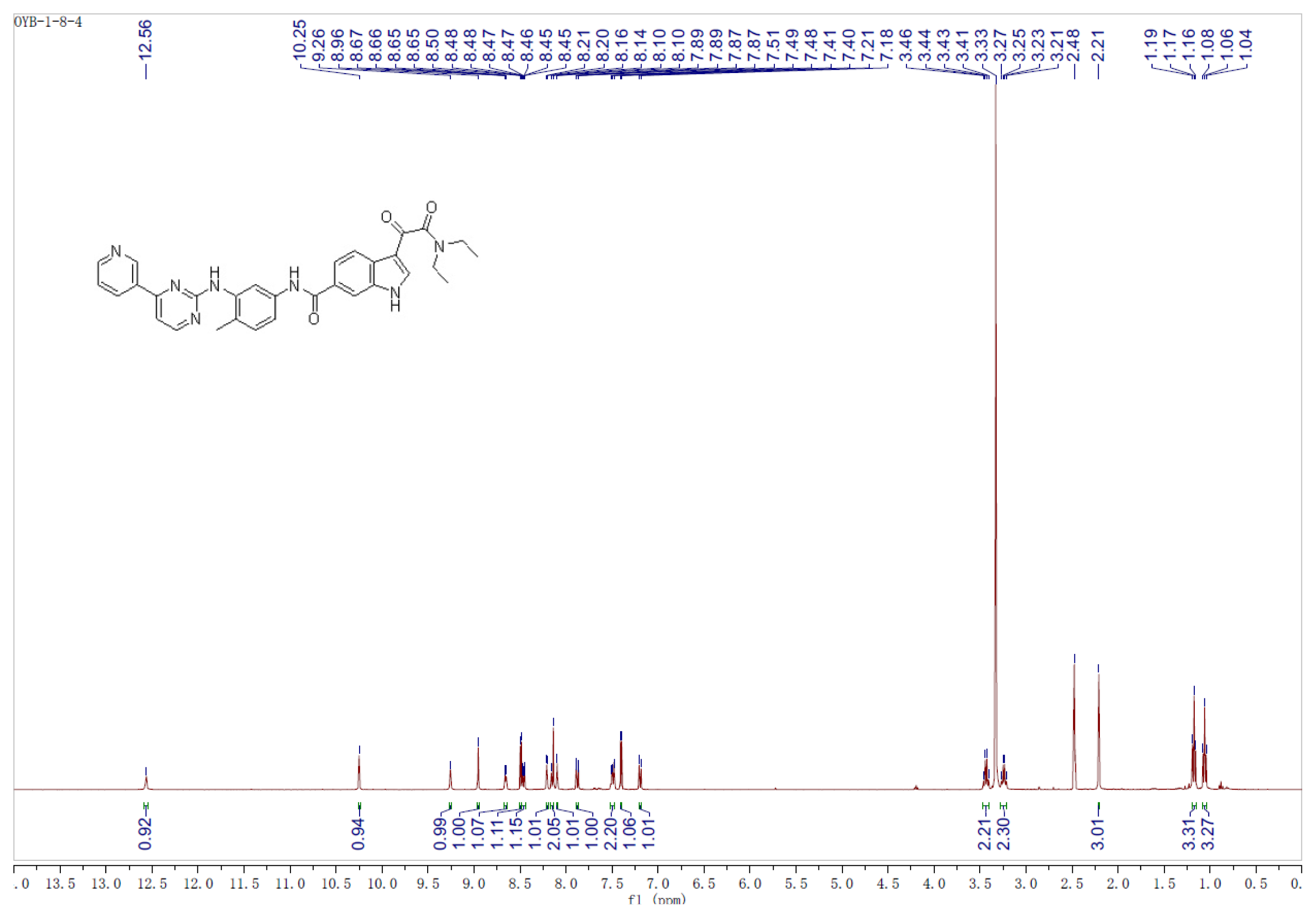

Supplement: Figure S7 — 1H-NMR spectrum of I4. [file turkjchem-47-2-426s7.tif]

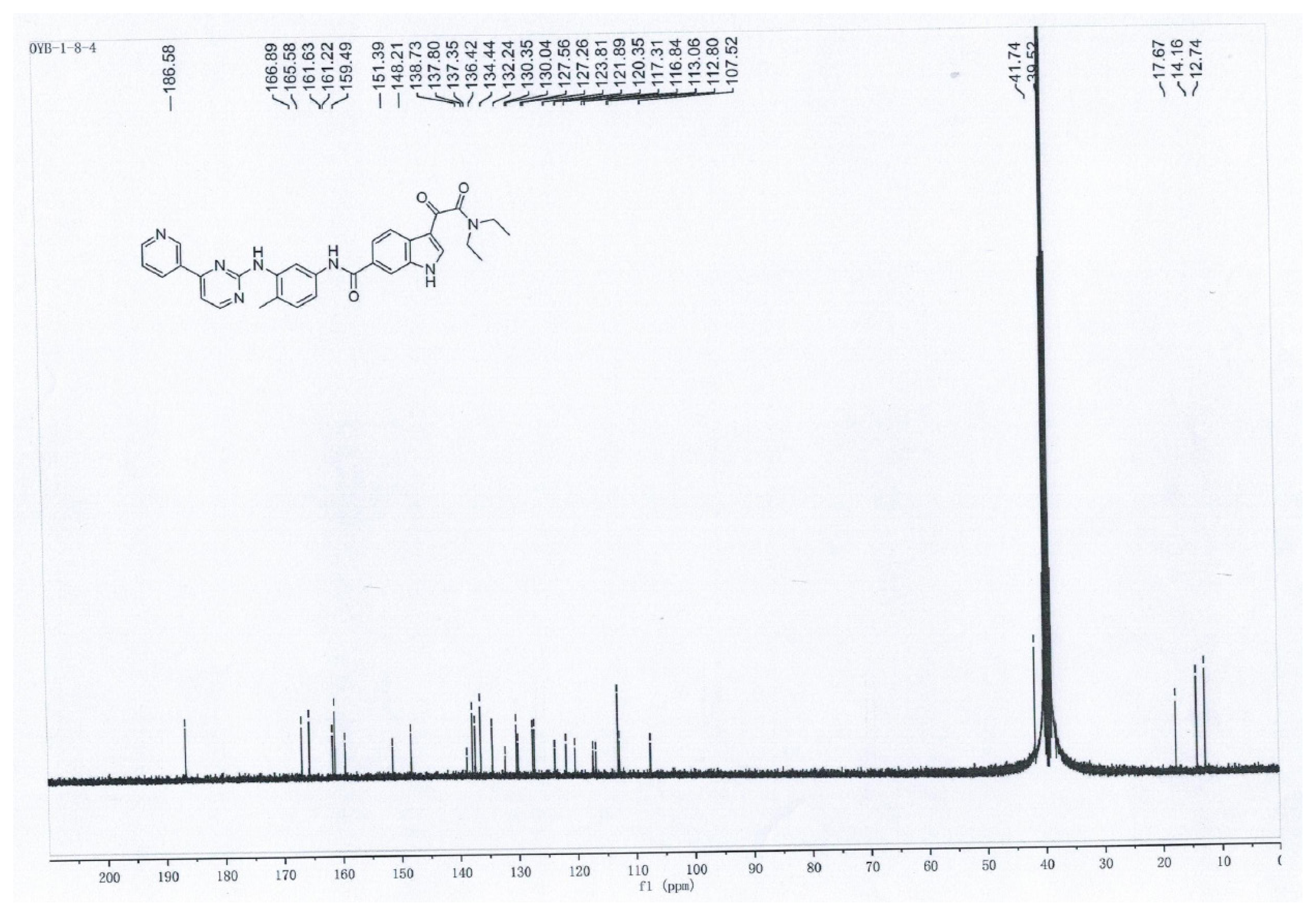

Supplement: Figure S8 — 13C-NMR spectrum of I4. [file turkjchem-47-2-426s8.tif]

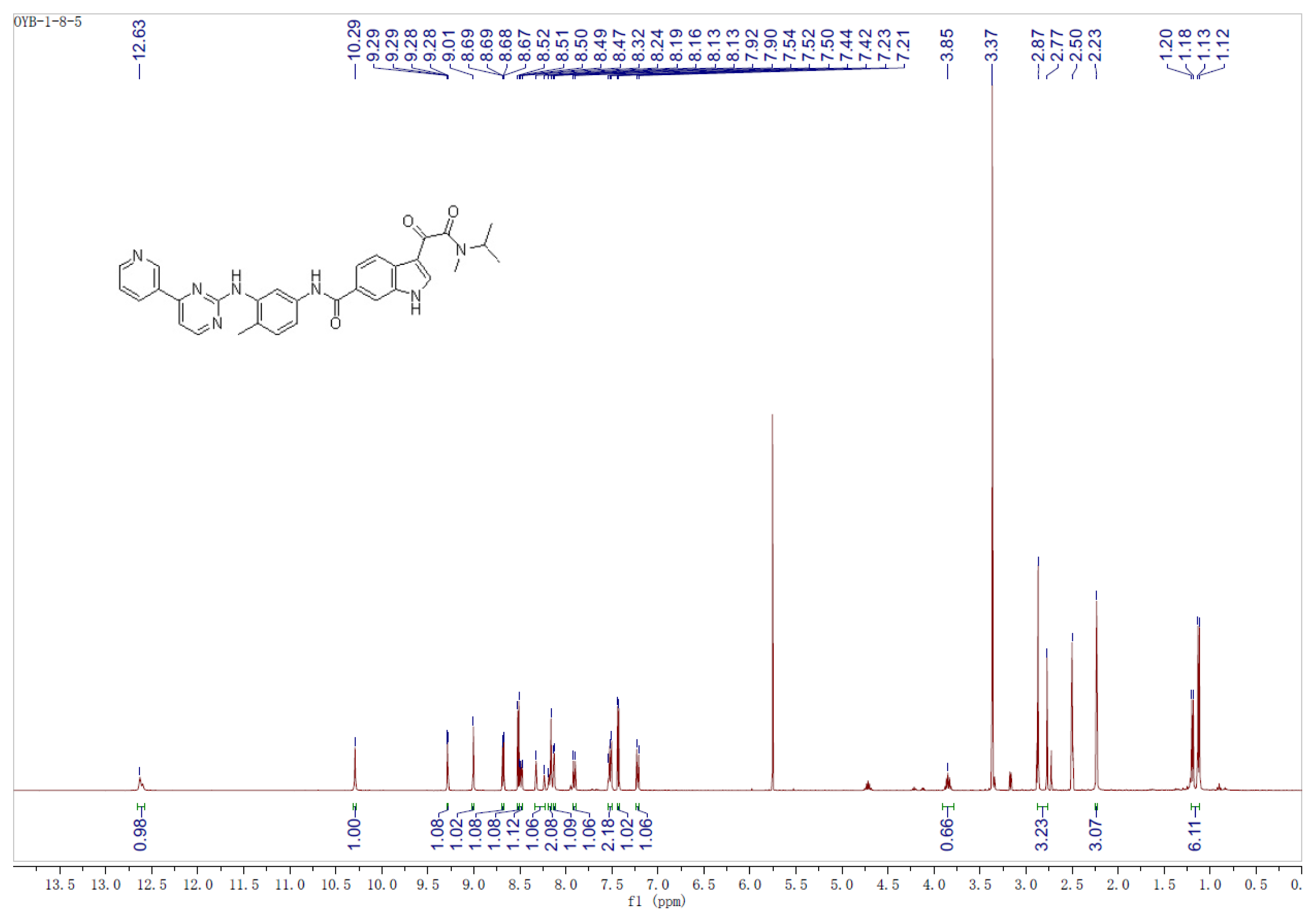

Supplement: Figure S9 — 1H-NMR spectrum of I5. [file turkjchem-47-2-426s9.tif]

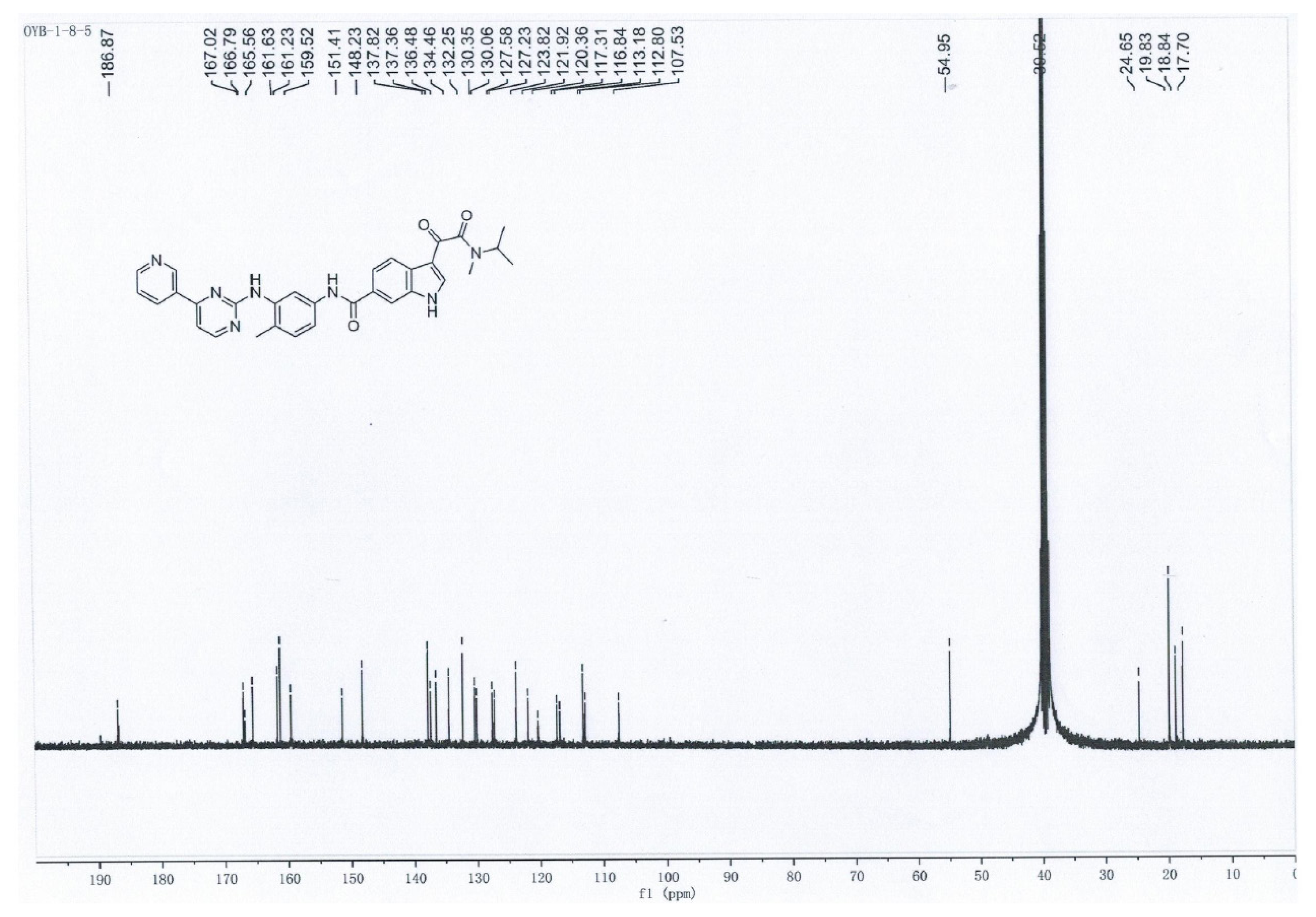

Supplement: Figure S10 — 13C-NMR spectrum of I5. [file turkjchem-47-2-426s10.tif]

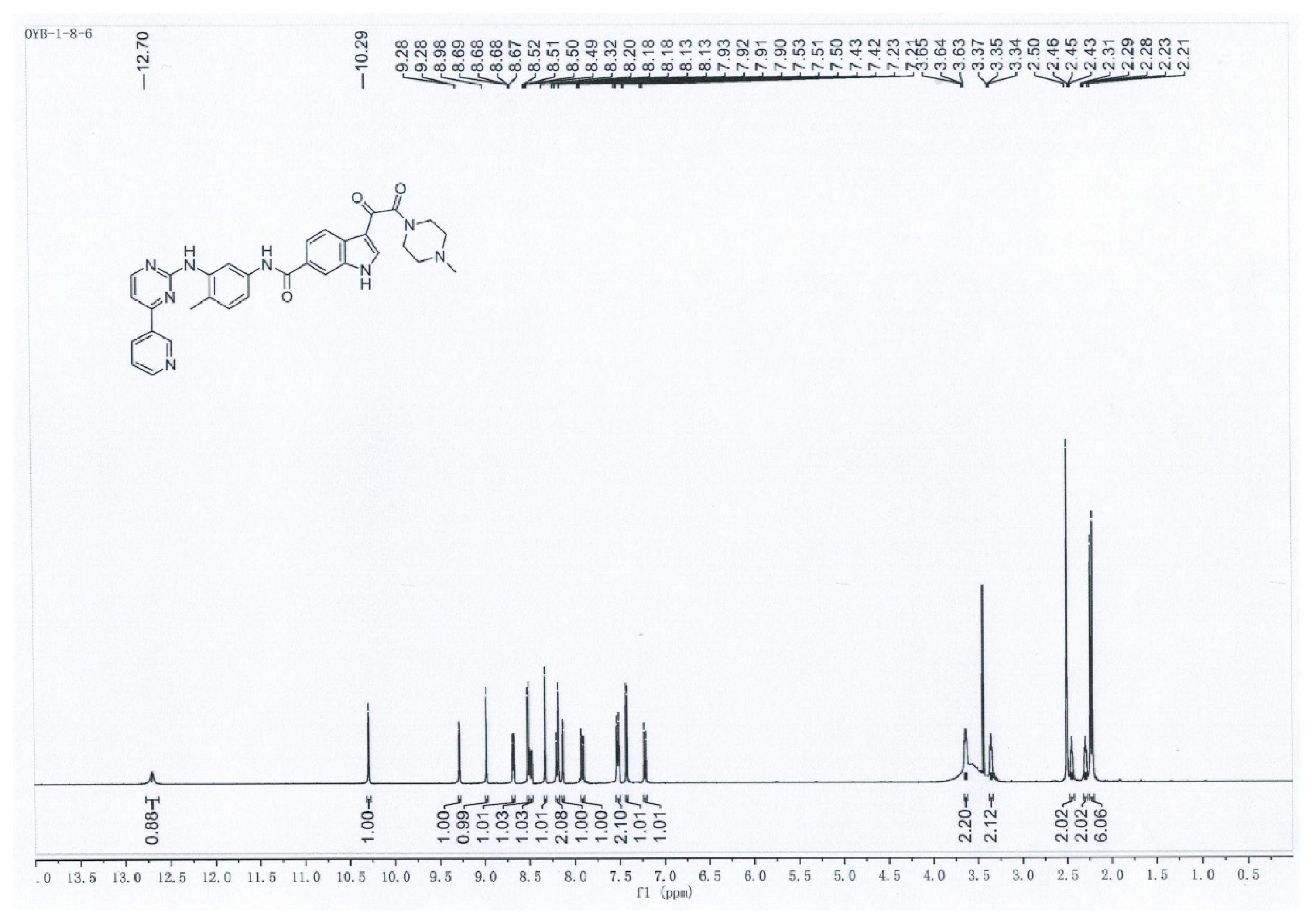

Supplement: Figure S11 — 1H-NMR spectrum of I6. [file turkjchem-47-2-426s11.tif]

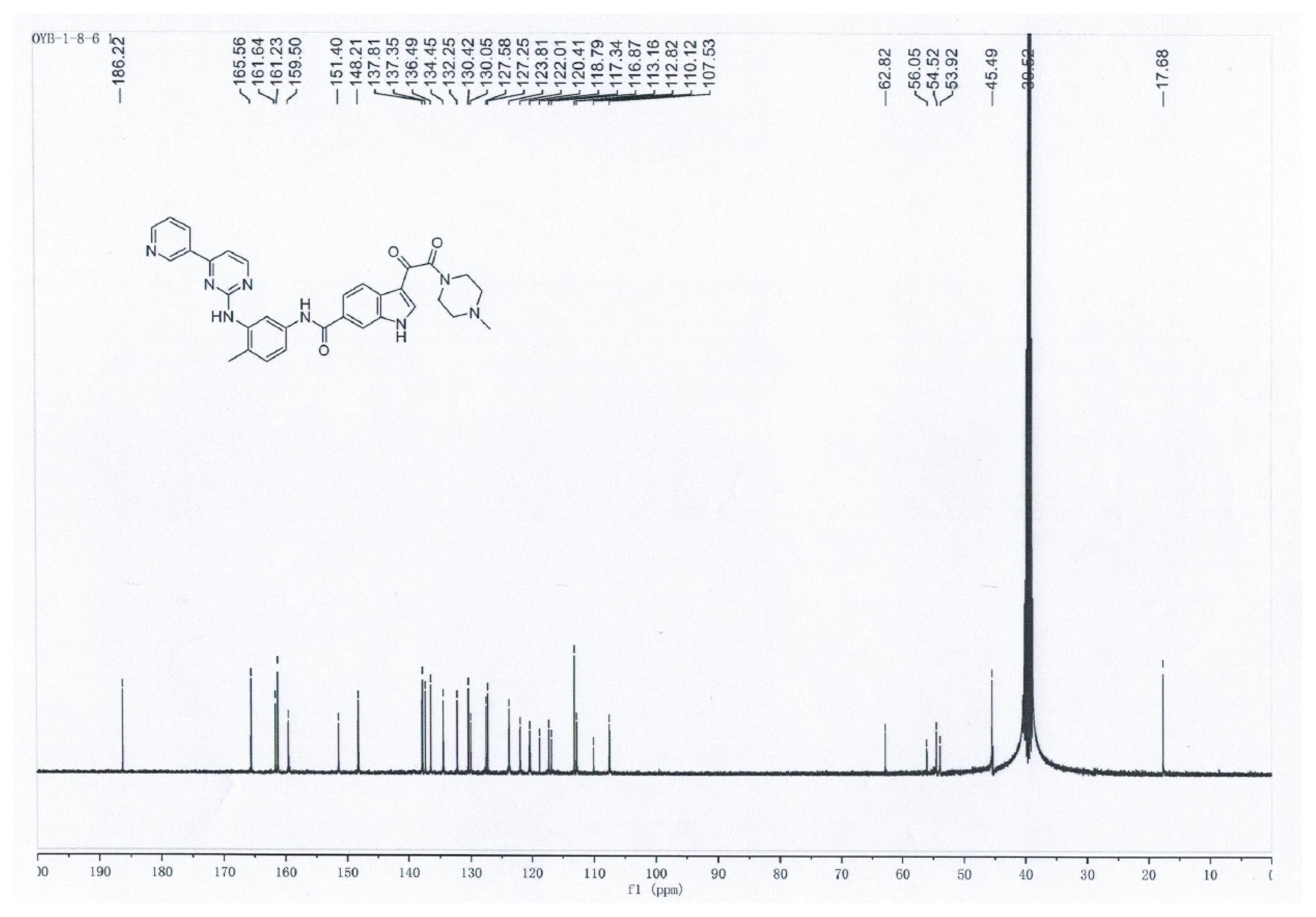

Supplement: Figure S12 — 13C-NMR spectrum of I6. [file turkjchem-47-2-426s12.tif]

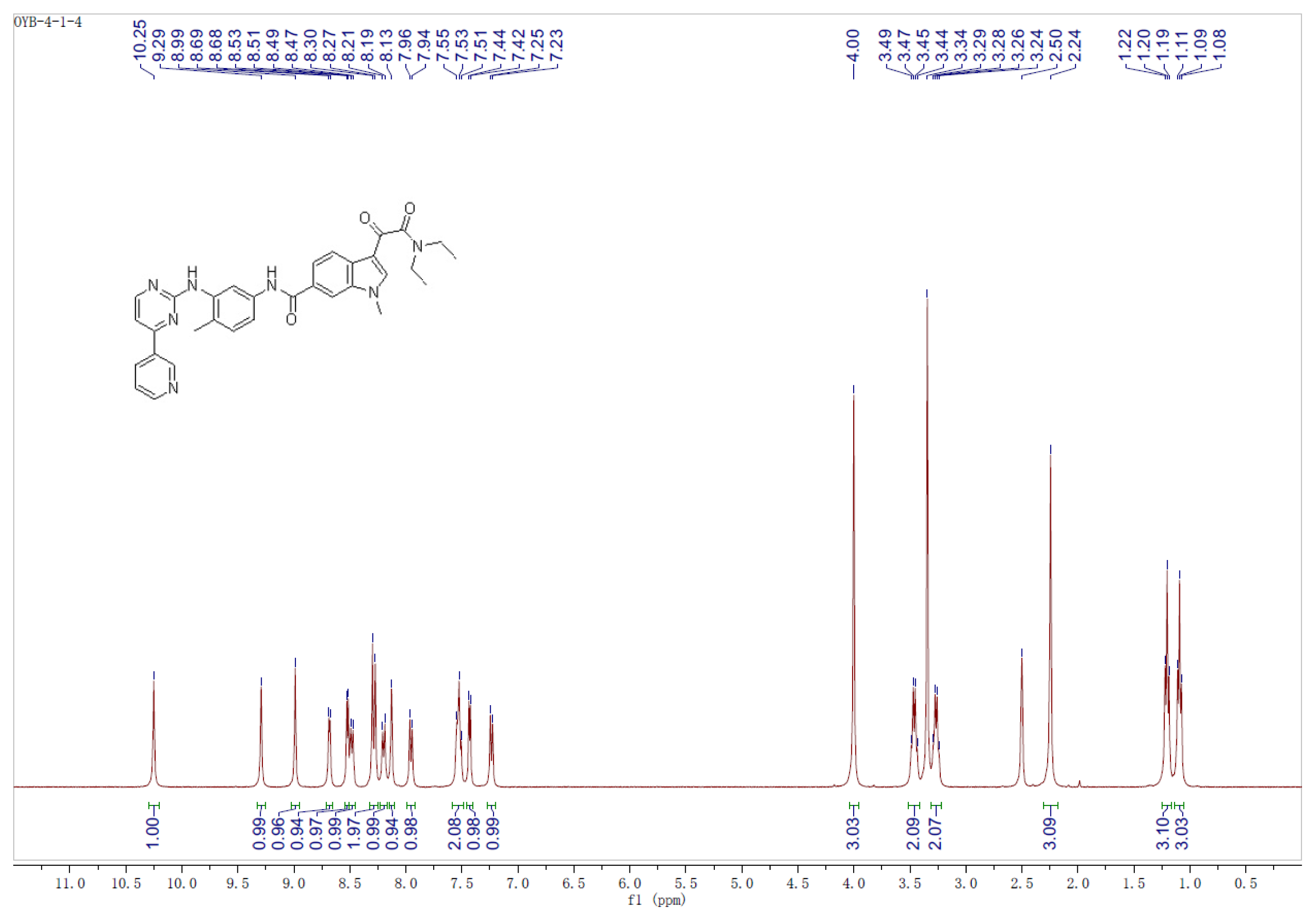

Supplement: Figure S13 — 1H-NMR spectrum of I7. [file turkjchem-47-2-426s13.tif]

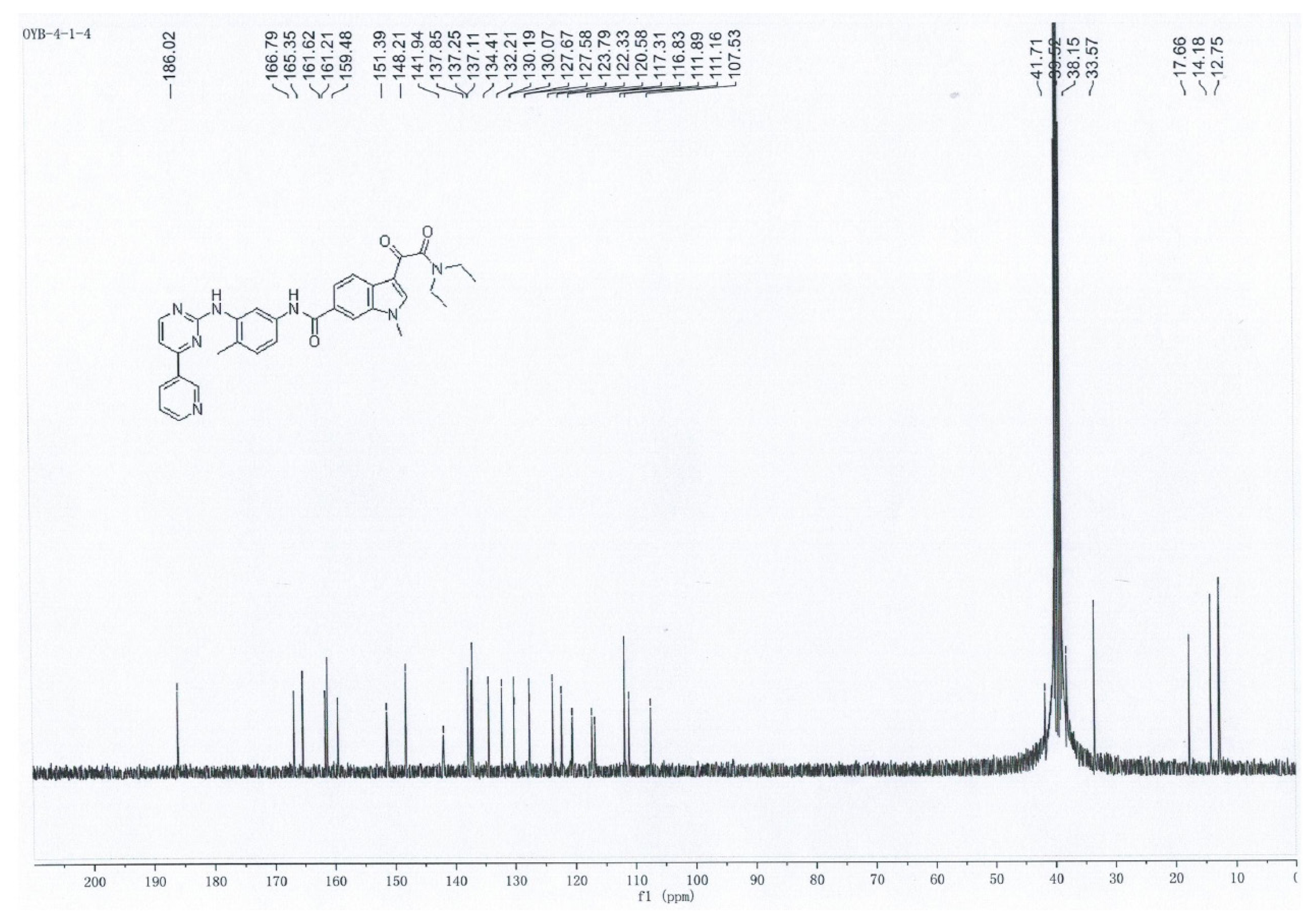

Supplement: Figure S14 — 13C-NMR spectrum of I7. [file turkjchem-47-2-426s14.tif]

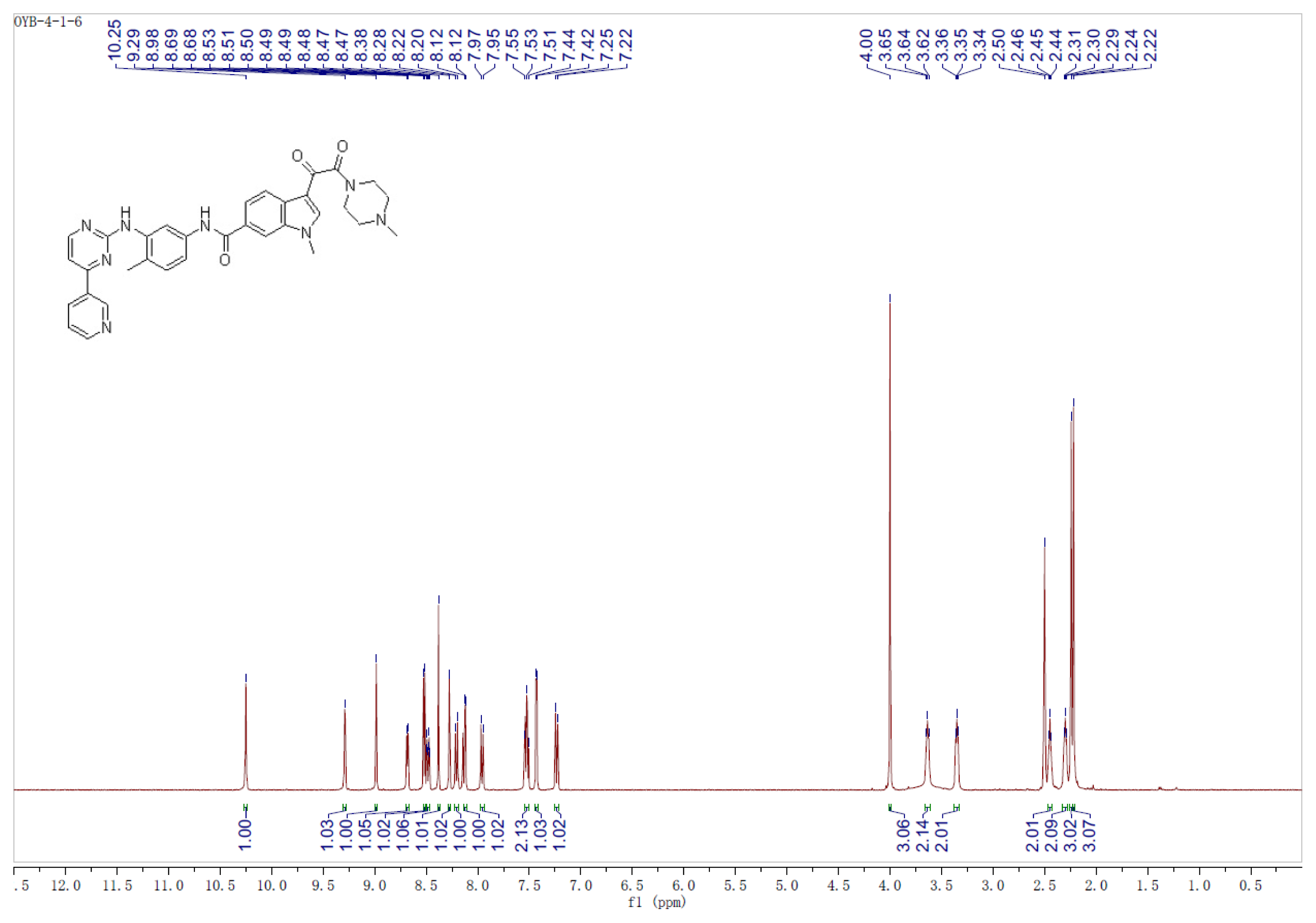

Supplement: Figure S15 — 1H-NMR spectrum of I8. [file turkjchem-47-2-426s15.tif]

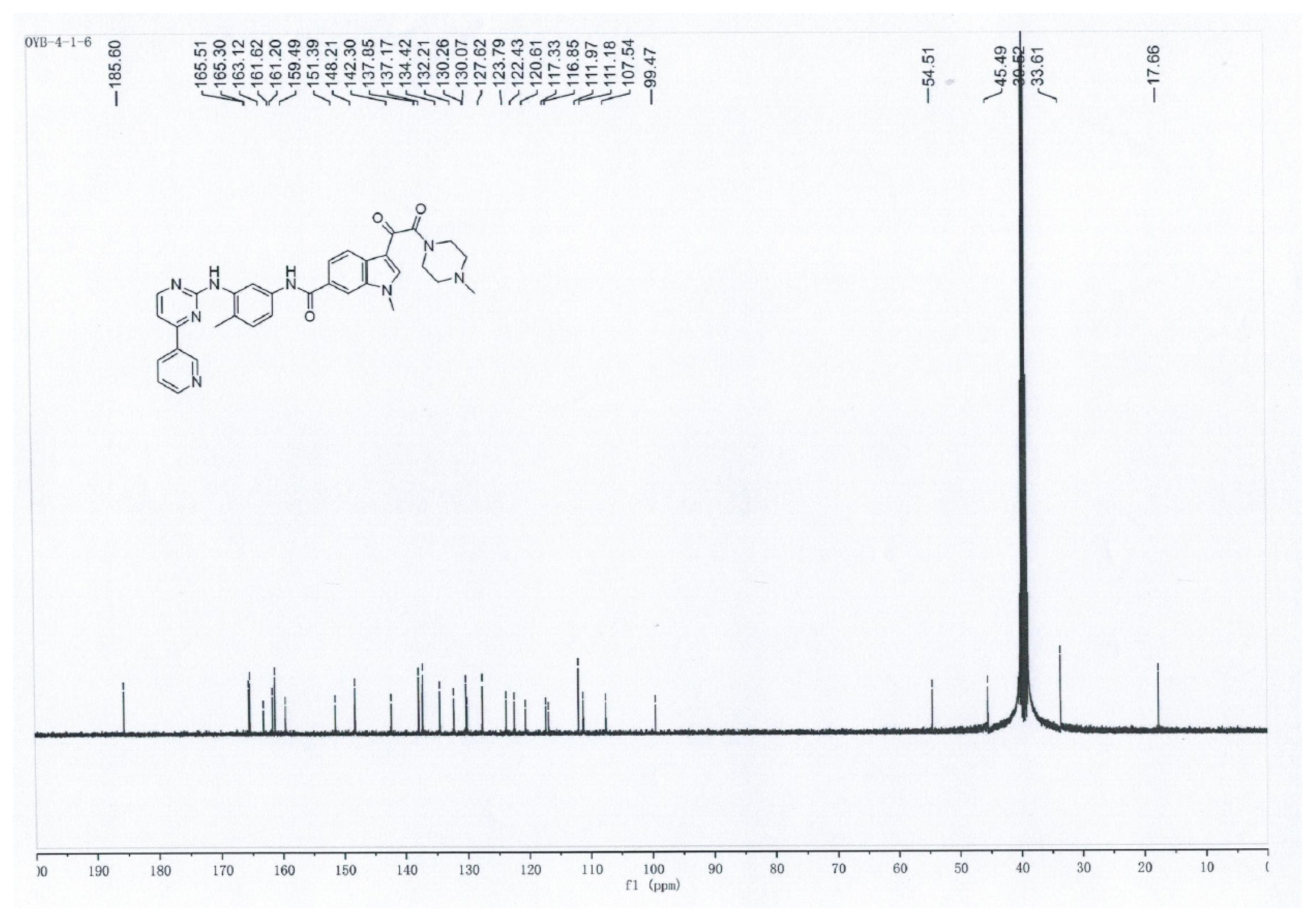

Supplement: Figure S16 — 13C-NMR spectrum of I8. [file turkjchem-47-2-426s16.tif]

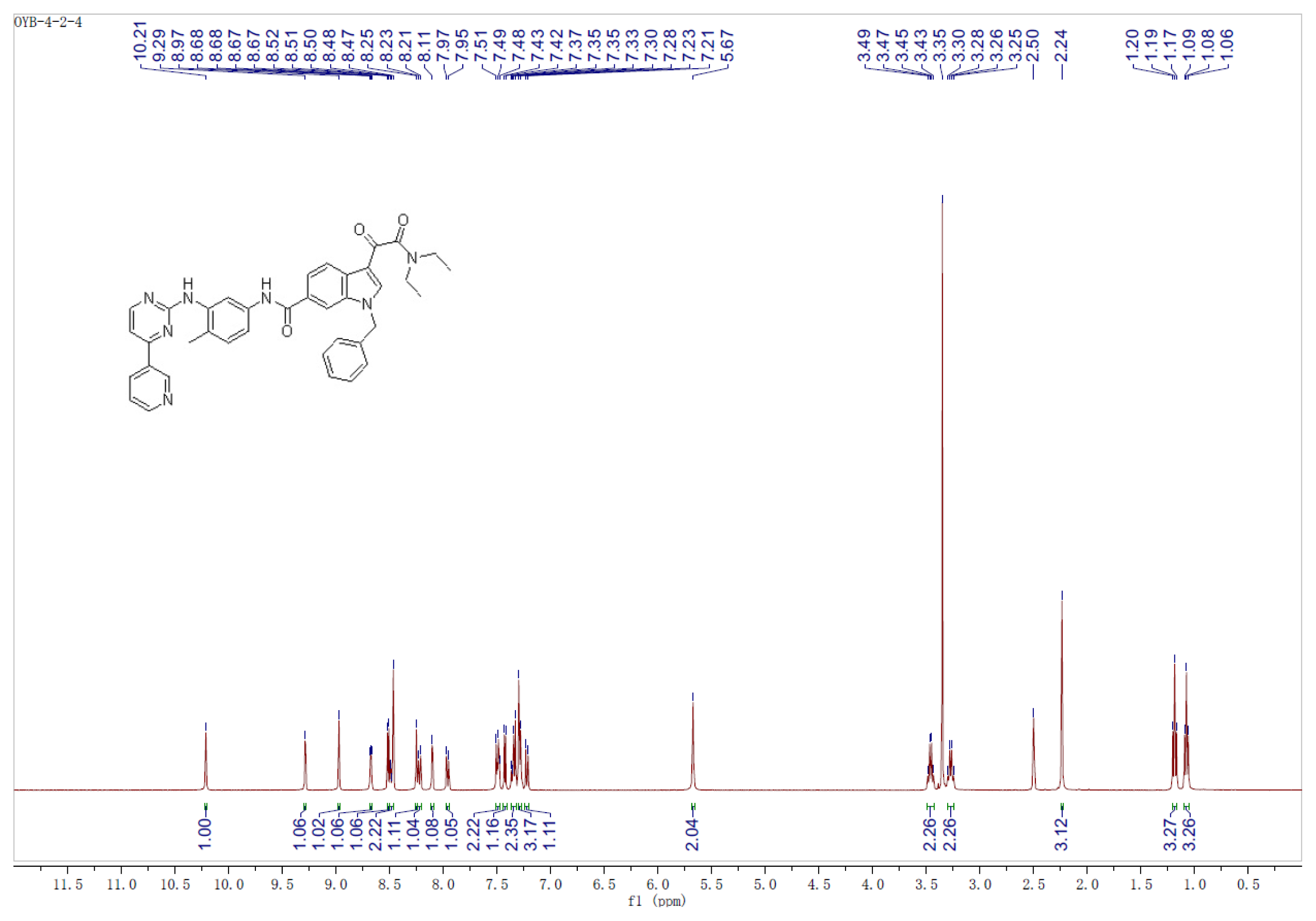

Supplement: Figure S17 — 1H-NMR spectrum of I9. [file turkjchem-47-2-426s17.tif]

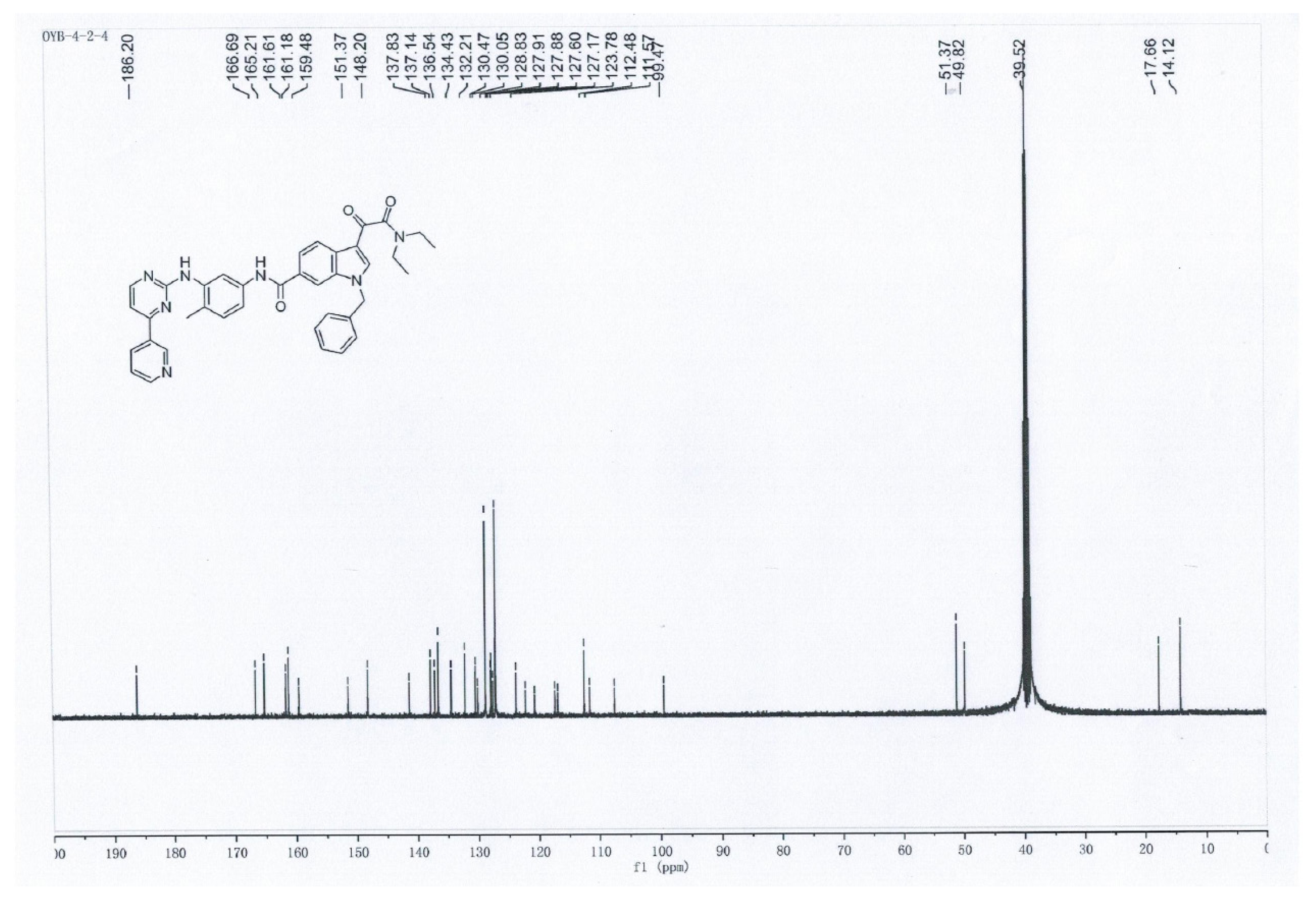

Supplement: Figure S18 — 13C-NMR spectrum of I9. [file turkjchem-47-2-426s18.tif]

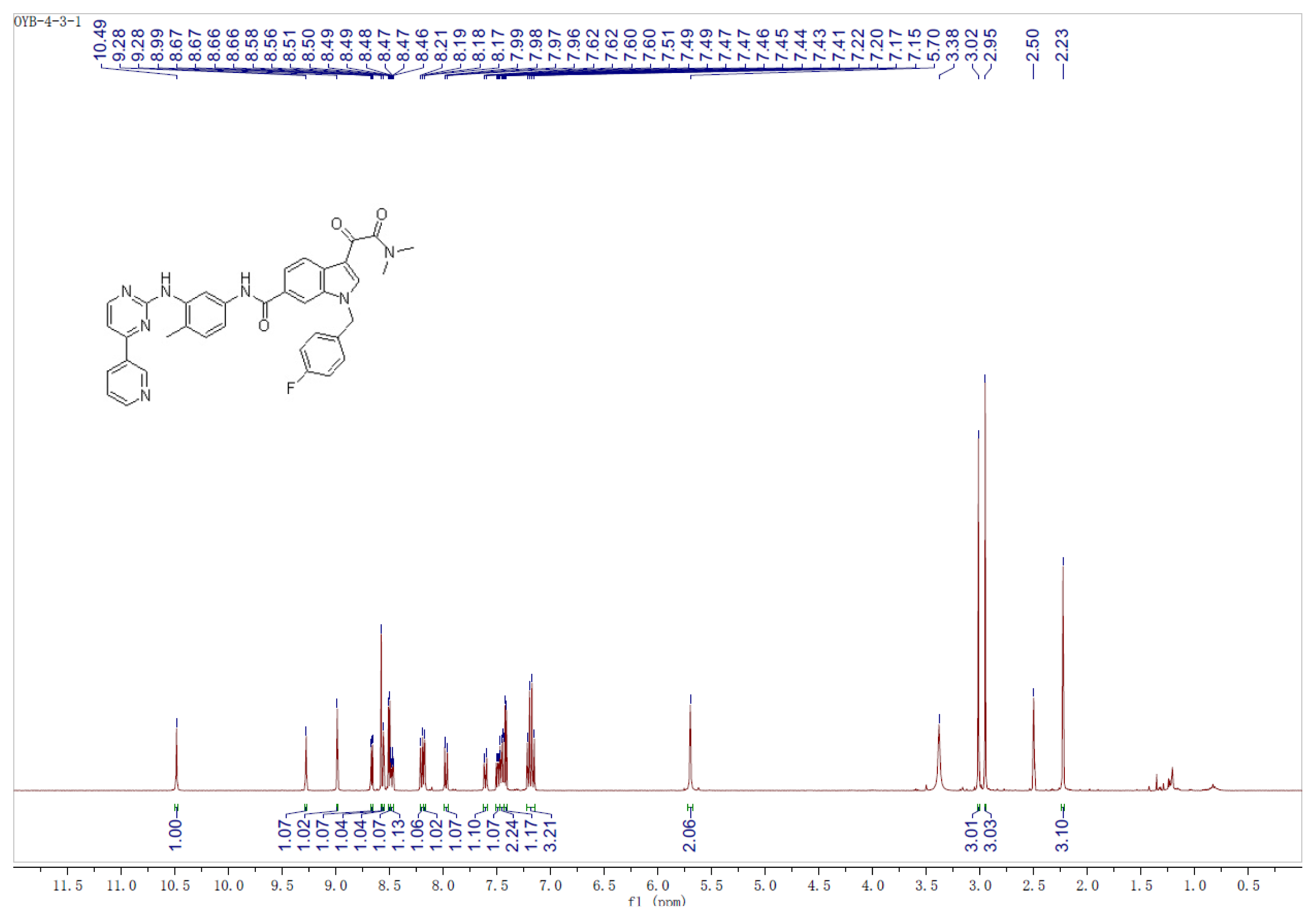

Supplement: Figure S19 — 1H-NMR spectrum of I10. [file turkjchem-47-2-426s19.tif]

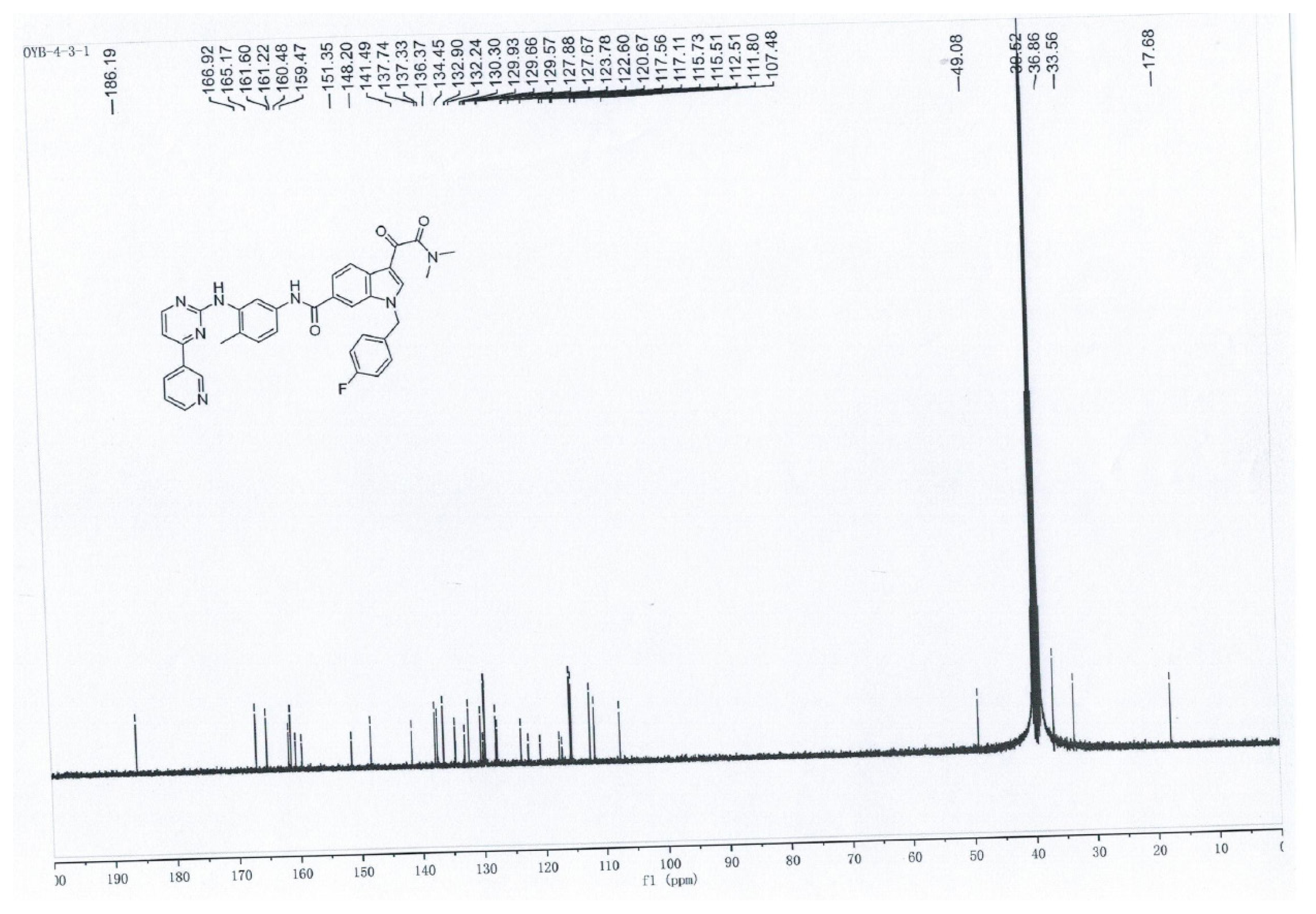

Supplement: Figure S20 — 13C-NMR spectrum of I10. [file turkjchem-47-2-426s20.tif]

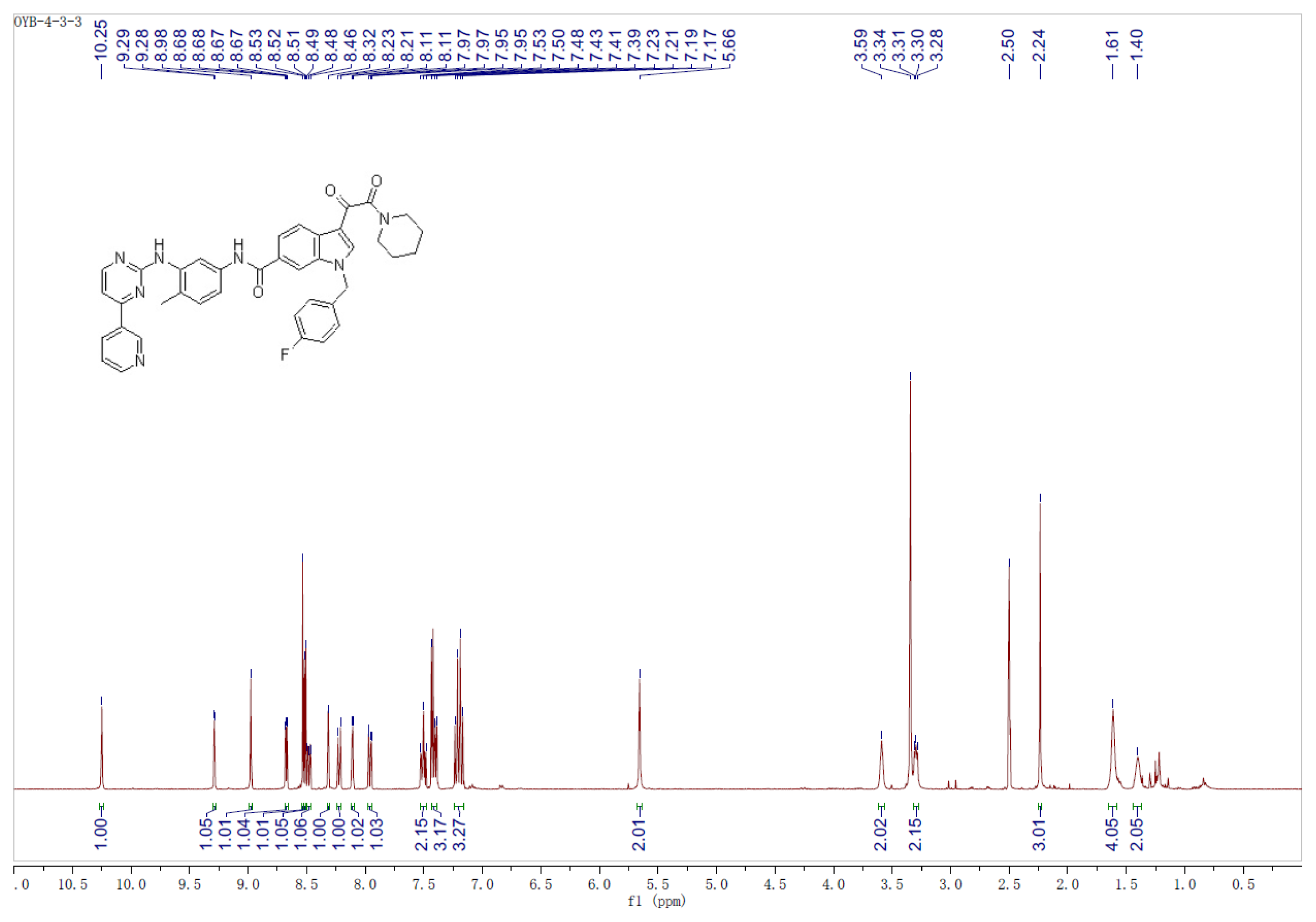

Supplement: Figure S21 — 1H-NMR spectrum of I11. [file turkjchem-47-2-426s21.tif]

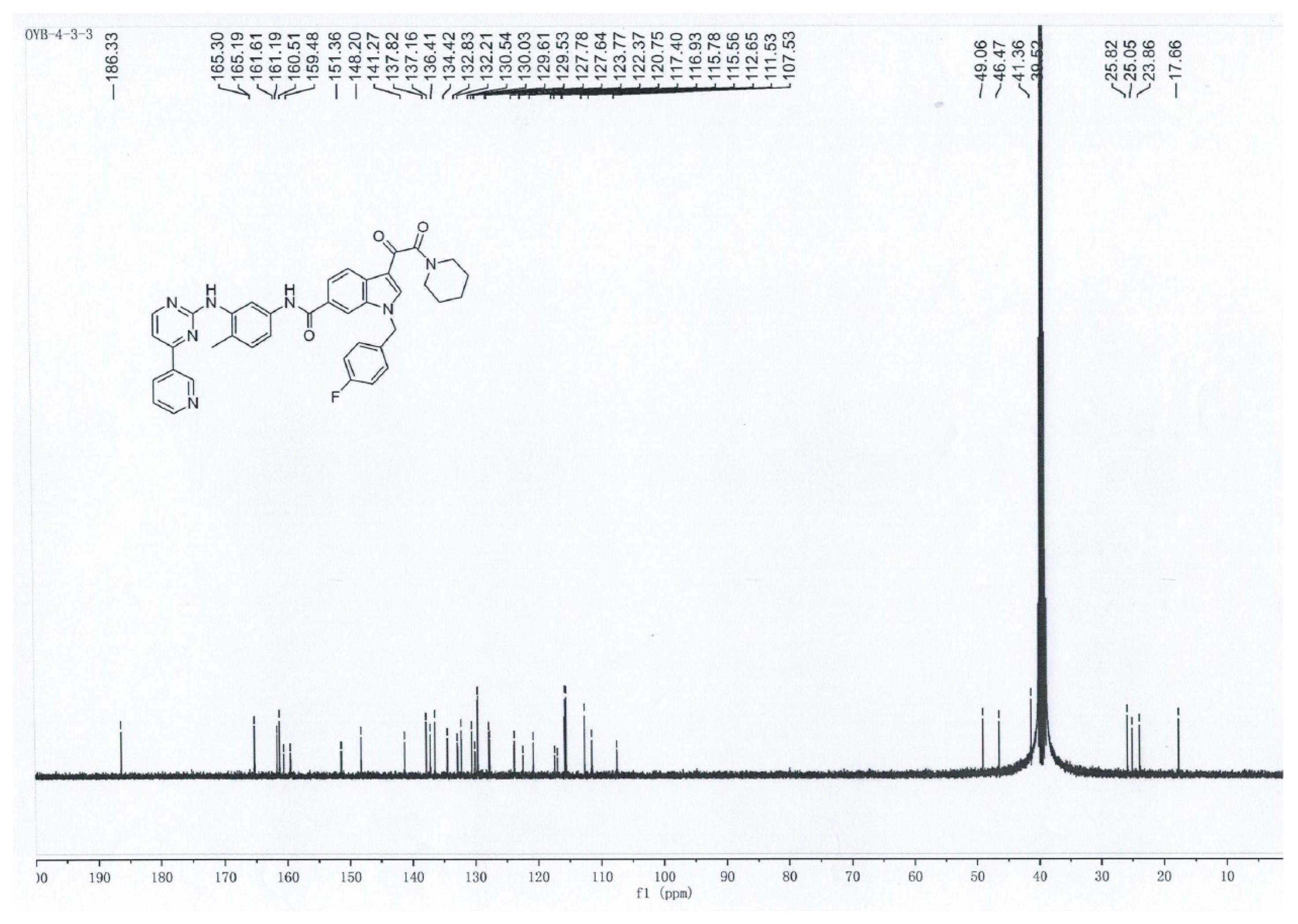

Supplement: Figure S22 — 13C-NMR spectrum of I11. [file turkjchem-47-2-426s22.tif]

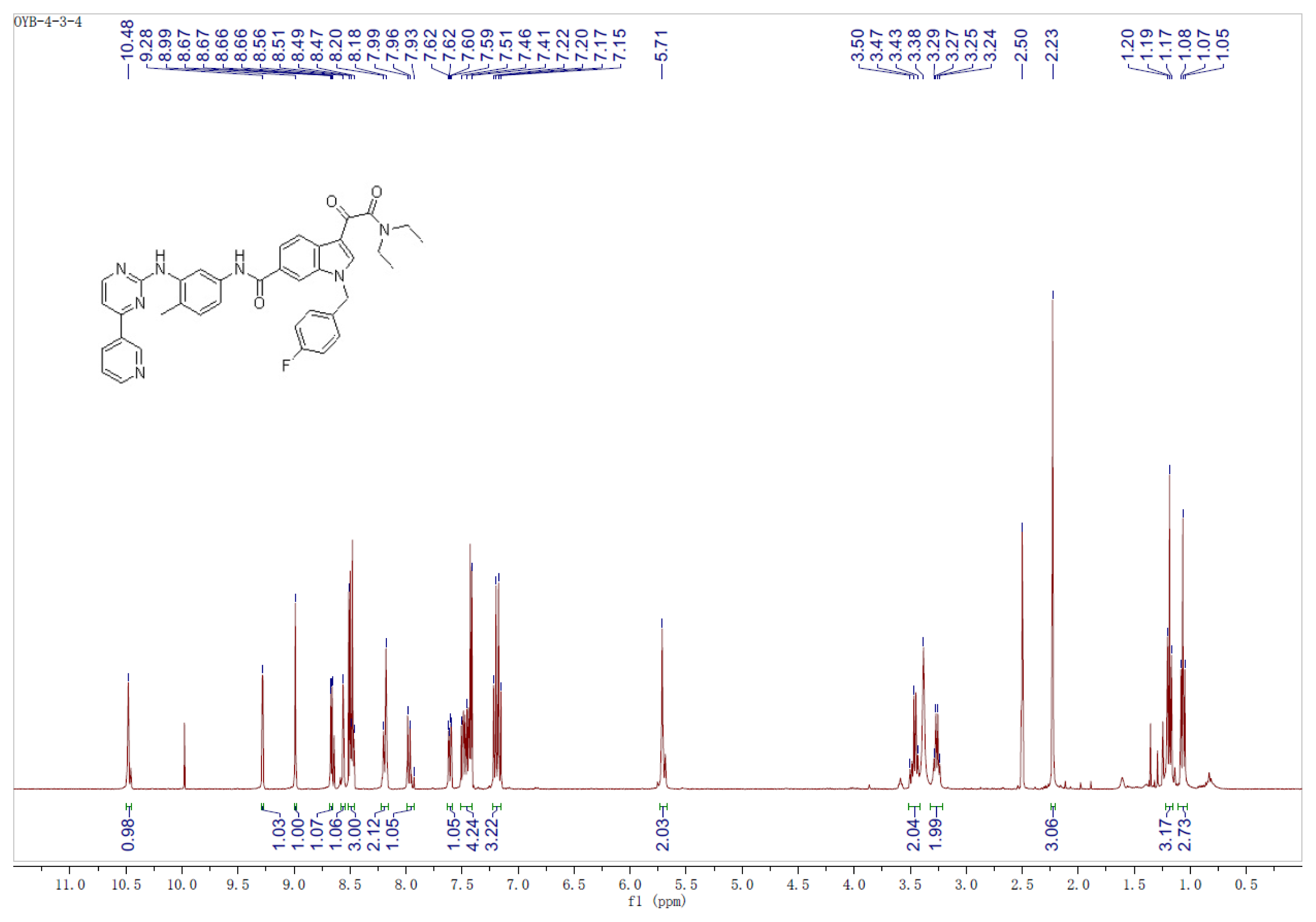

Supplement: Figure S23 — 1H-NMR spectrum of I12. [file turkjchem-47-2-426s23.tif]

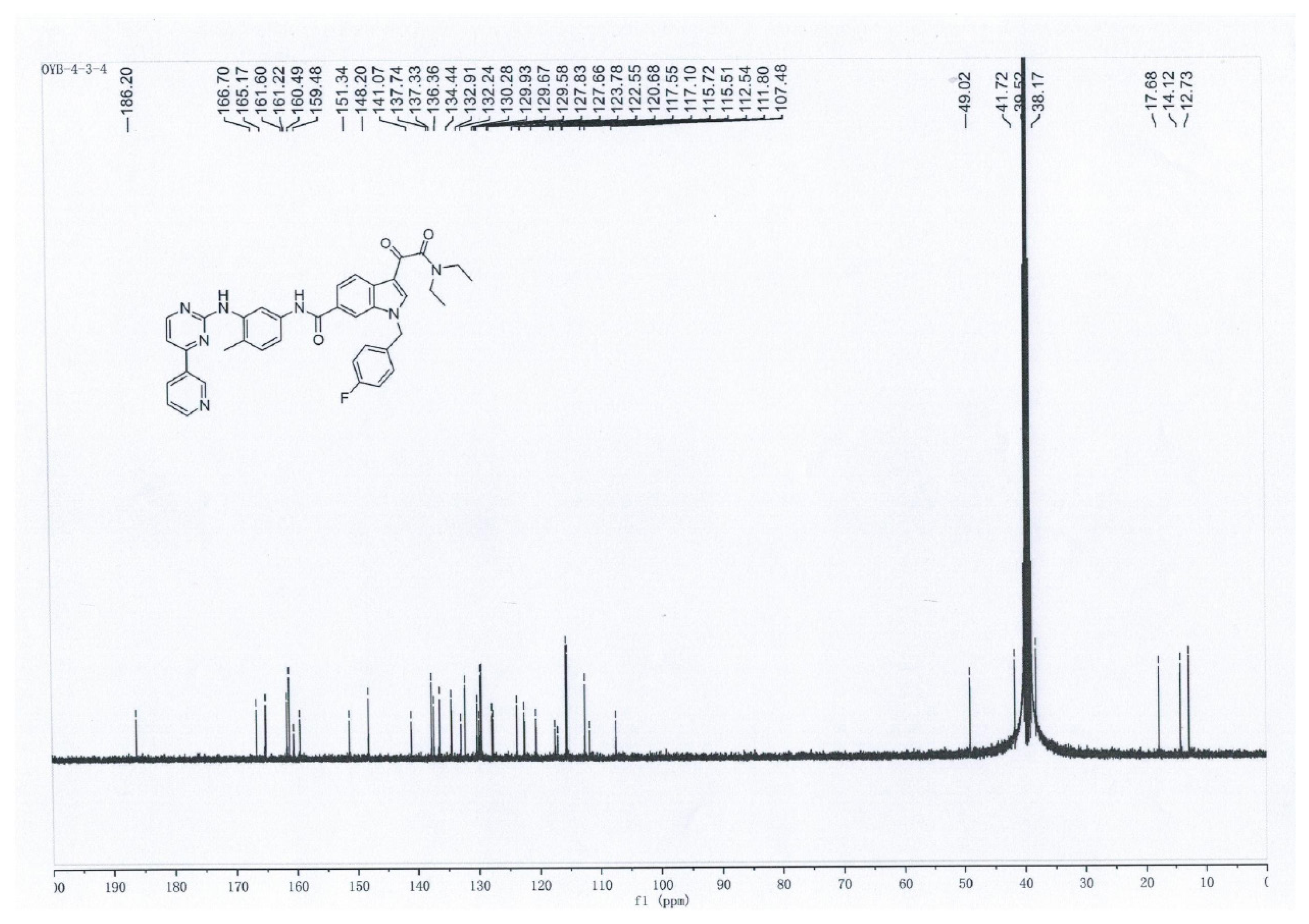

Supplement: Figure S24 — 13C-NMR spectrum of I12. [file turkjchem-47-2-426s24.tif]

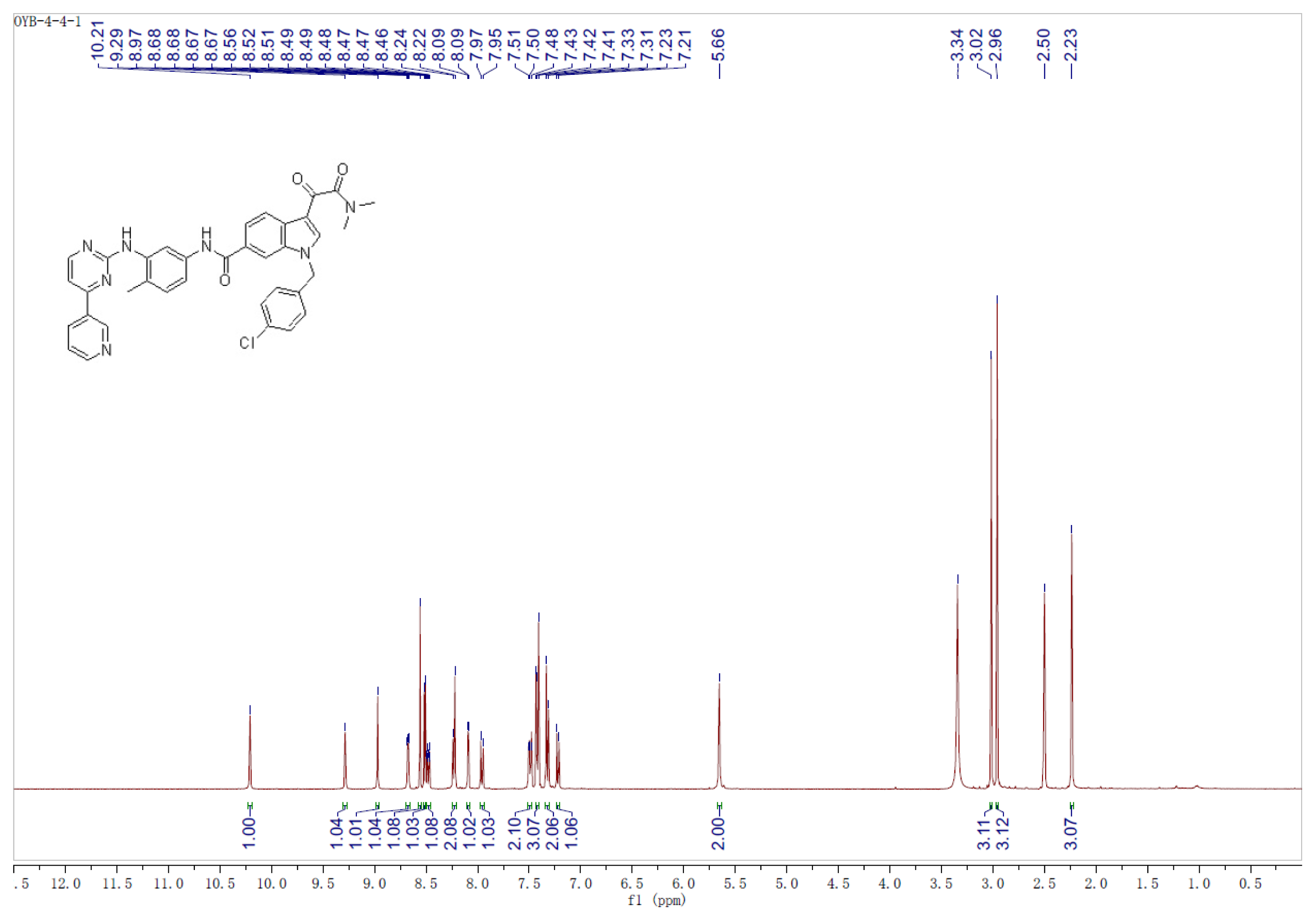

Supplement: Figure S25 — 1H-NMR spectrum of I13. [file turkjchem-47-2-426s25.tif]

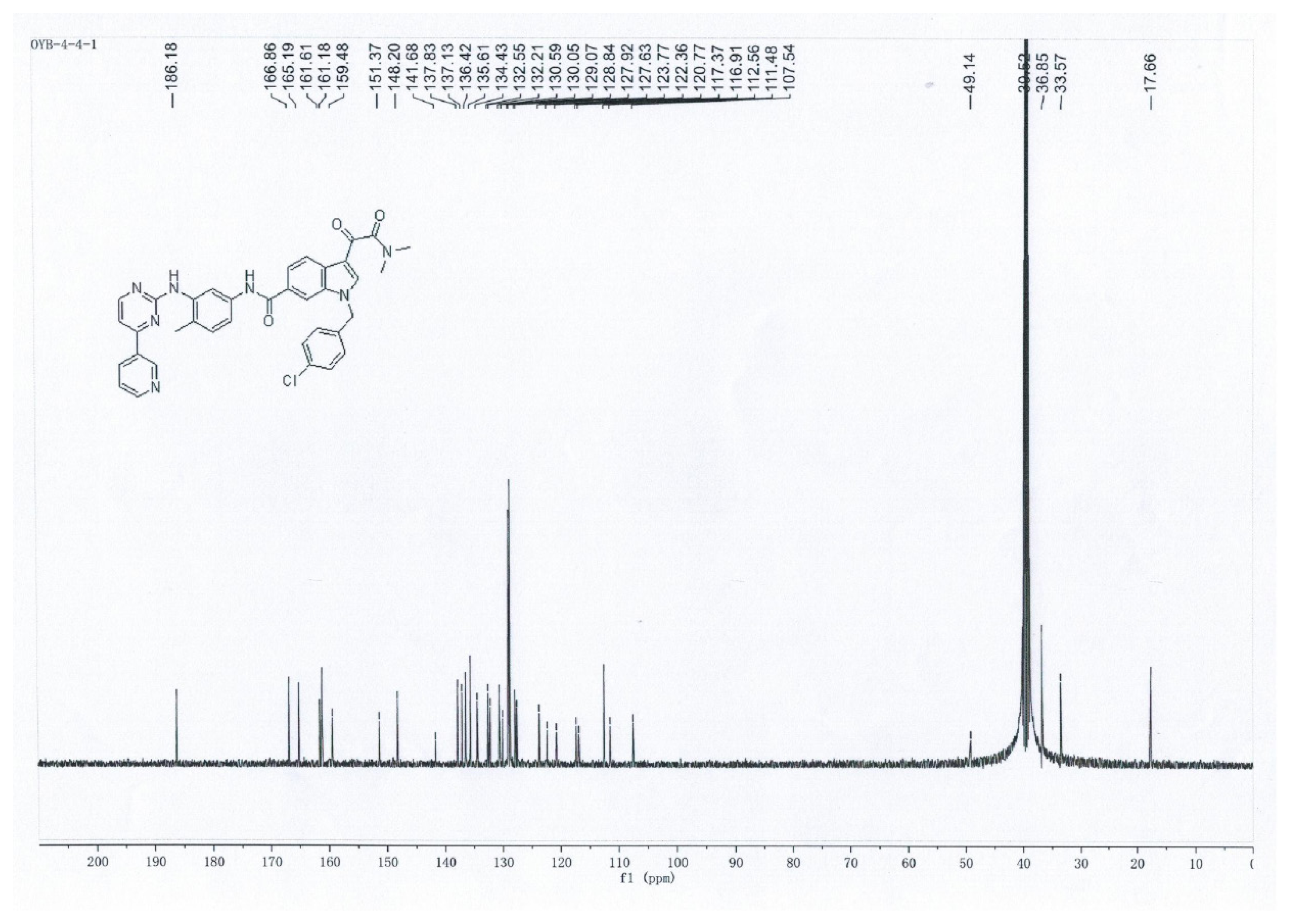

Supplement: Figure S26 — 13C-NMR spectrum of I13. [file turkjchem-47-2-426s26.tif]

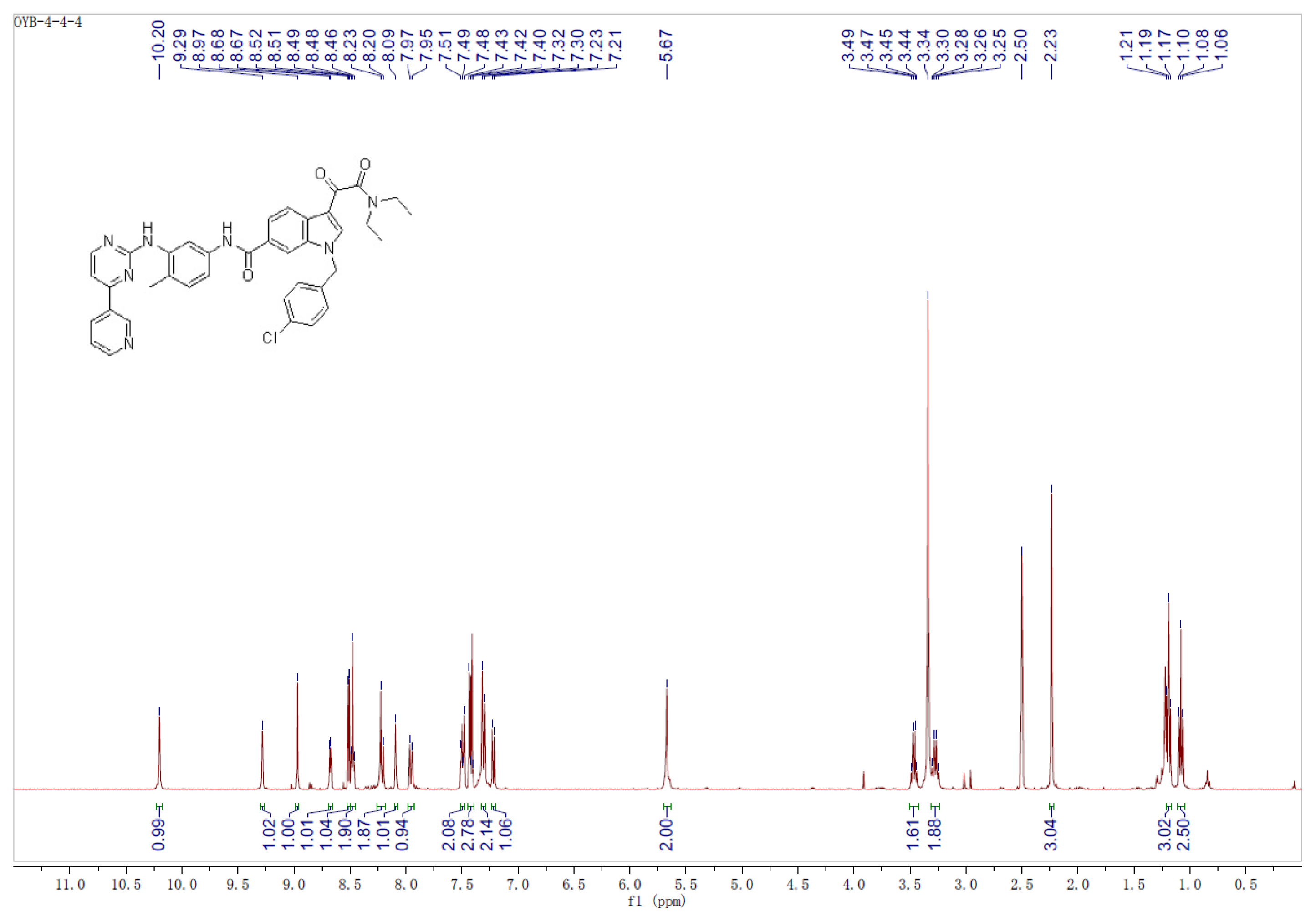

Supplement: Figure S27 — 1H-NMR spectrum of I14. [file turkjchem-47-2-426s27.tif]

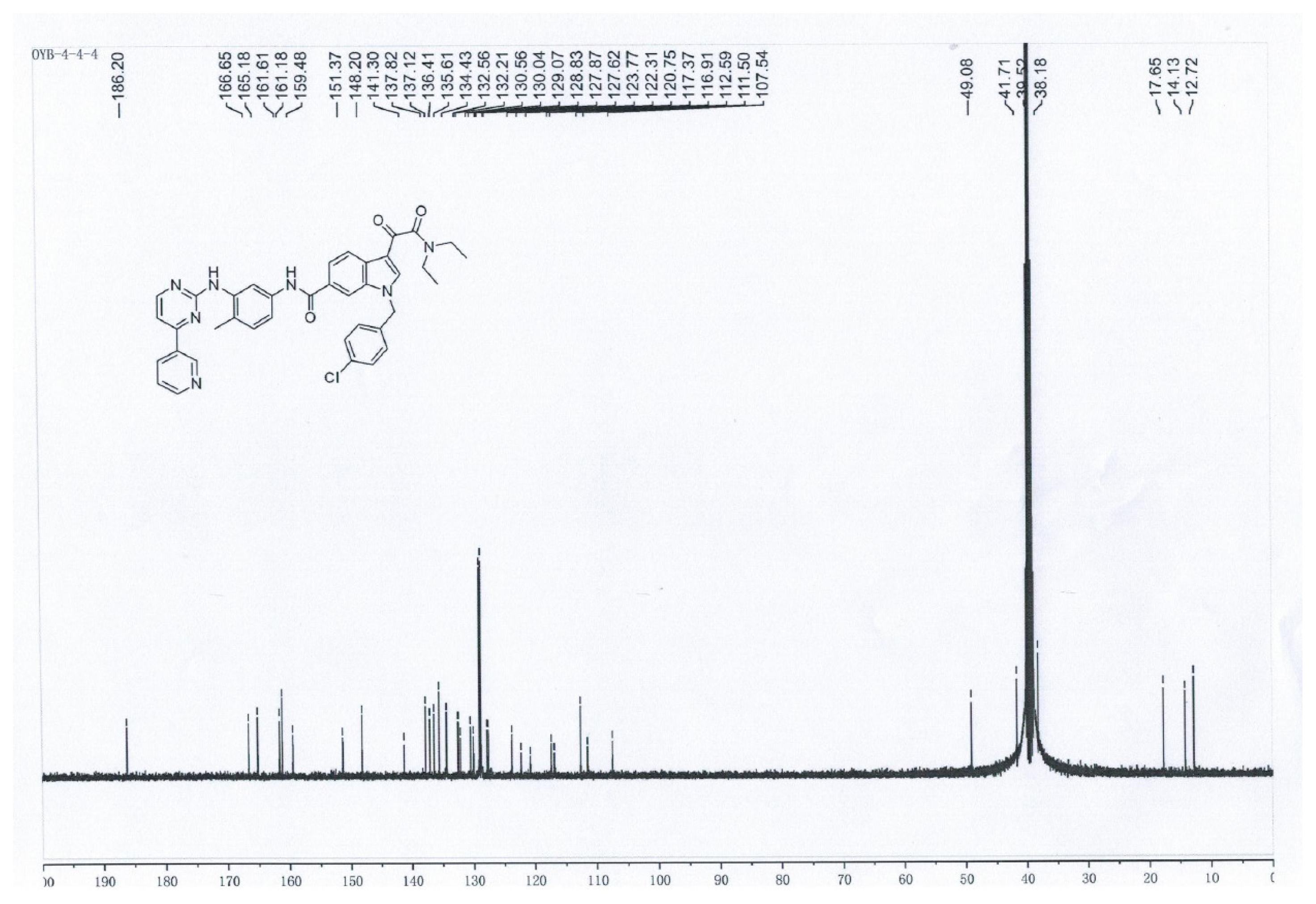

Supplement: Figure S28 — 13C-NMR spectrum of I14. [file turkjchem-47-2-426s28.tif]

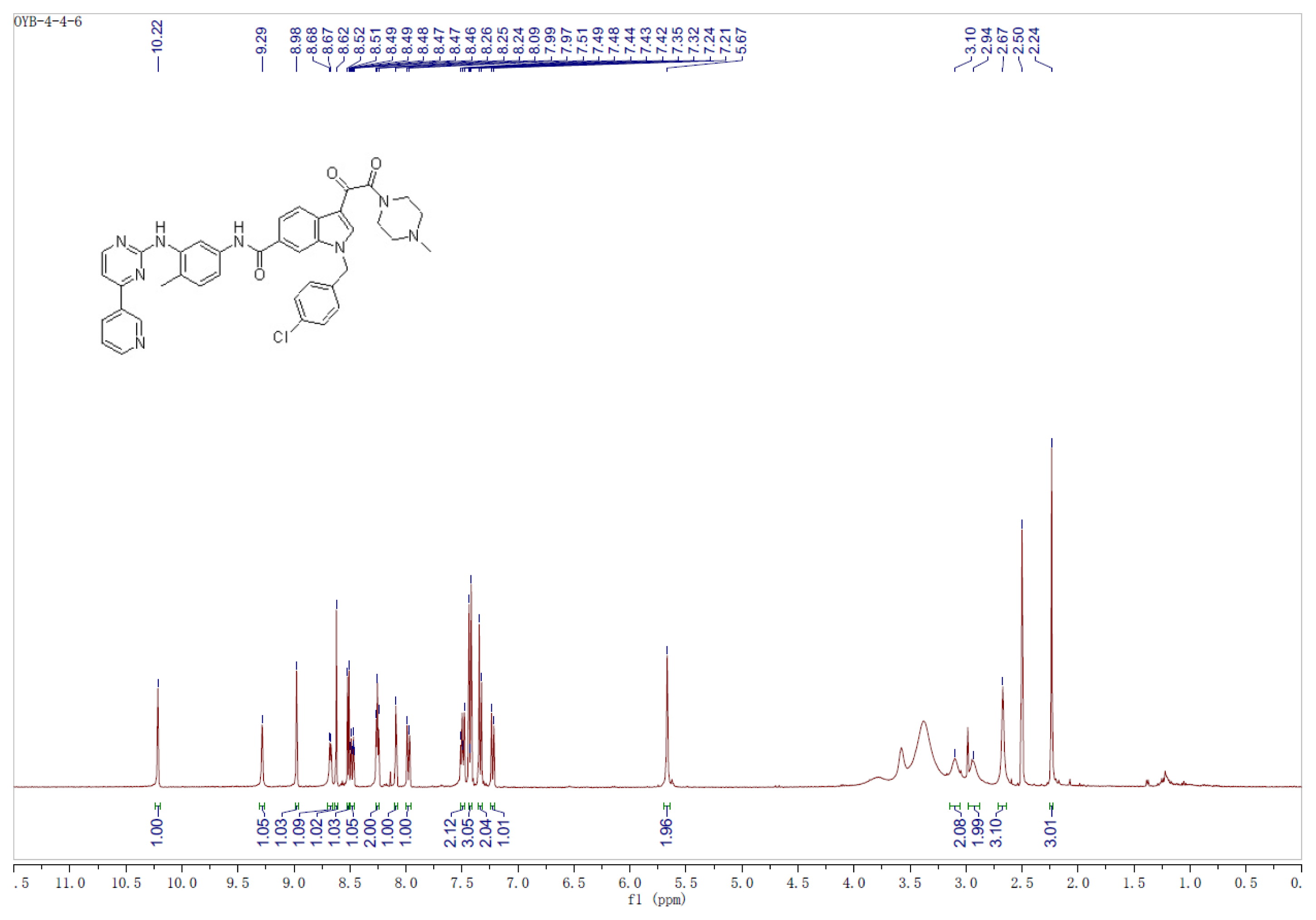

Supplement: Figure S29 — 1H-NMR spectrum of I15. [file turkjchem-47-2-426s29.tif]

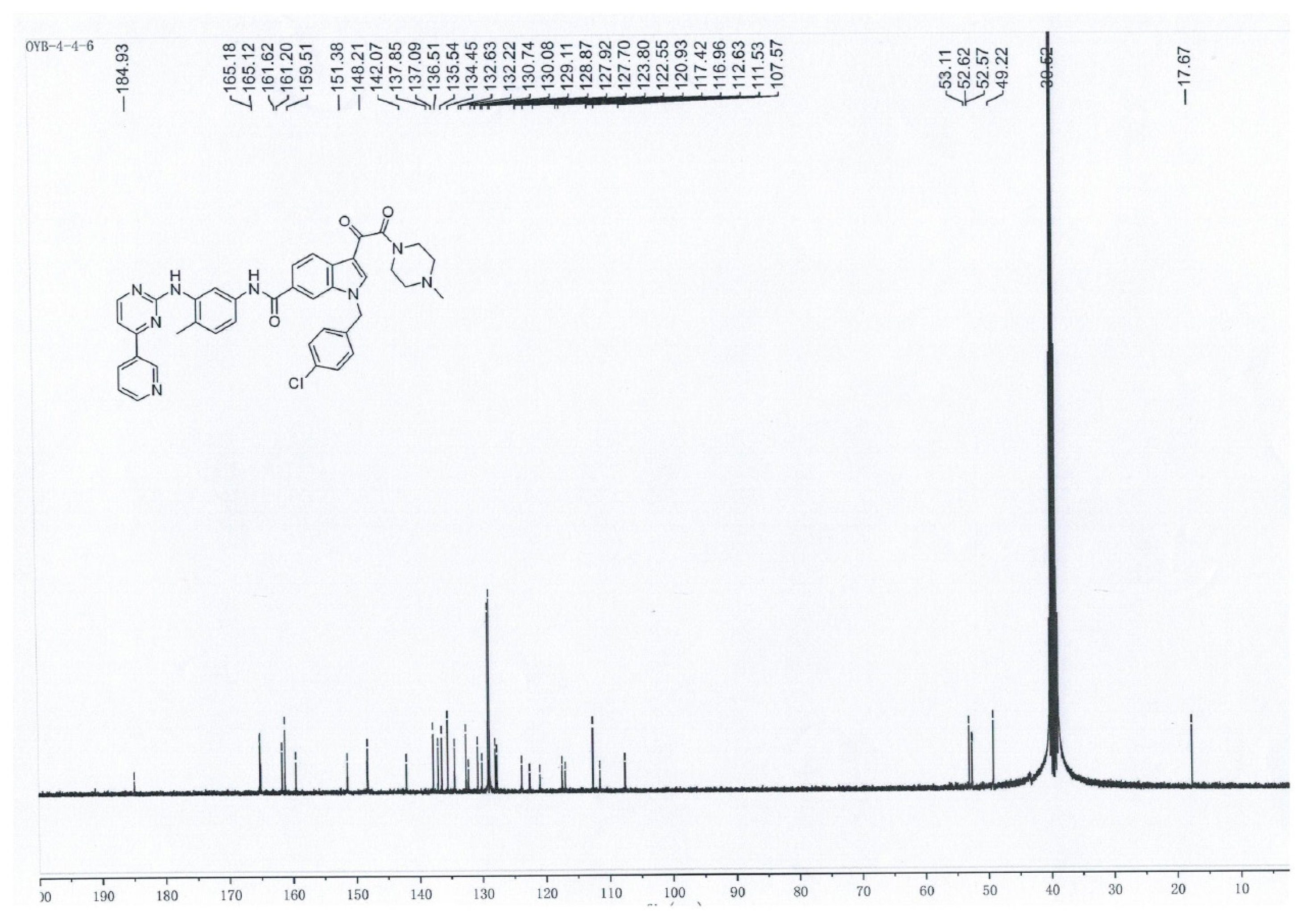

Supplement: Figure S30 — 13C-NMR spectrum of I15. [file turkjchem-47-2-426s30.tif]
